# Internet-Based Telerehabilitation Versus in-Person Therapeutic Exercises in Young Adult Females With Chronic Neck Pain and Forward Head Posture: Randomized Controlled Trial

**DOI:** 10.2196/74979

**Published:** 2025-07-25

**Authors:** Patcharin Nilmart, Arrada Sichuai, Asree Chedang, Chanikarn Goontharo, Nollapan Janjamsai

**Affiliations:** 1 Faculty of Physical Therapy, Srinakharinwirot University, 63 Moo 7 Rangsit-Nakhon Nayok Highway, Ongkharak, Nakhon Nayok, 26120, Thailand, 66 89-4635274; 2 School of Allied Health Sciences, Walailak University, Nakhon Si Thammarat, Thailand

**Keywords:** telerehabilitation, chronic neck pain, forward head posture, therapeutic exercise, videoconferencing supervision

## Abstract

**Background:**

Neck pain is a common musculoskeletal disorder, often linked to forward head posture (FHP). Studies have shown that exercise interventions can improve pain, craniovertebral angle (CVA), range of motion, and function in individuals with FHP. While telerehabilitation exercise has proven effective for other musculoskeletal conditions, its effectiveness in addressing neck pain and FHP is still being investigated.

**Objective:**

This study aimed to evaluate and compare the effectiveness of an internet-based telerehabilitation therapeutic exercise program with an in-person supervised program in improving clinical outcomes among young adult females with chronic nonspecific neck pain and FHP. The study hypothesized that there would be no significant differences in outcomes between the 2 groups.

**Methods:**

A randomized controlled trial was conducted with 50 participants experiencing chronic neck pain and FHP, recruited through public announcement and voluntary sign-up. Participants were randomly assigned to either a telerehabilitation group or an in-person supervision group. Both groups completed the same 6-week, physiotherapist-supervised therapeutic exercise program, delivered via Zoom (Zoom Video Communications) or in the physiotherapy department laboratory, respectively. Outcome assessments were conducted face-to-face by blinded assessors at baseline, after 4 and 6 weeks of intervention, and at a 2-week follow-up. Outcome measures included pain intensity (assessed using the Visual Analog Scale [VAS]), CVA, neck disability (assessed using the Neck Disability Index [NDI]), and cervical range of motion (CROM). Adherence was monitored using attendance logs.

**Results:**

Of the 50 participants, 48 completed the intervention with 1 dropout from each group. Adherence among completers was 100 percent in both groups. All 50 participants were included in the analysis using the intention-to-treat principle. No differences in effectiveness were found between the telerehabilitation and in-person groups, as no significant interaction effect between group and time was observed across all outcome measures including VAS, CVA, NDI, and CROM (*P* values ranged .07-.61). However, improvements were observed in all outcomes across time, including a 2.2‐ to 4.1-cm reduction in VAS, 5°-8.8° increase in CVA, 3.3‐ to 7.1-point reduction in NDI (*P*<.001 for all), and 3.5°-22.7° increase in CROM (*P*<.001 to *P*=.04).

**Conclusions:**

Both telerehabilitation and in-person supervision were similarly effective in improving pain, posture, neck disability, and CROM in young adult females with chronic neck pain and FHP. These findings suggest that telerehabilitation may be a feasible and accessible alternative to conventional in-person therapeutic exercise programs for managing chronic neck pain with FHP.

## Introduction

Neck pain is one of the most common musculoskeletal disorders and represents a significant public health concern within the general population [1]. Approximately 50% of people experience an essential episode of neck pain at some point in their lives [2]. Notably, female adolescents are particularly susceptible to persistent neck pain [3]. Neck pain is becoming more common due to the widespread use of smartphones [4]. This behavior is associated with increased activity in the neck extensor, erector spinae, and upper trapezius muscles, as well as increased head flexion, tilt angles, and forward head posture (FHP) [5]. This persistent tension on the cervical spine may lead to alterations in spinal curvature. Furthermore, research has linked head and neck posture to neck pain [6], with a negative correlation observed between neck pain and the craniovertebral angle (CVA) [7].

FHP is characterized by hyperextension of the upper cervical spine and increased flexion of the lower cervical and upper thoracic spines. This posture causes the head to shift forward from the gravity line in the sagittal plane [8,9]. Muscle imbalances result from such abnormal spinal alignment, with the deep cervical flexors, rhomboids, lower trapezius, and serratus anterior muscles becoming weakened or lengthened, while the upper trapezius, pectoralis, suboccipital, and levator scapulae muscles become shortened [10-12]. A previous study found that 60% of individuals with neck pain demonstrated FHP [13].

Several studies have reported significant improvements in pain, CVA, neck range of motion (ROM), and function in patients with FHP following in-person exercise interventions [14-16]. The usefulness of telemedicine and telerehabilitation in meeting health care demands during emergencies has become more widely acknowledged by medical professionals since the COVID-19 pandemic. According to recent data, the effectiveness of telerehabilitation, especially for physical therapists, may influence clinical decision-making and improve patient outcomes during and after the pandemic [17]. Previous studies have demonstrated the effectiveness of telerehabilitation for various musculoskeletal conditions, such as patellofemoral pain [18], knee osteoarthritis [19], low back pain [20], musculoskeletal shoulder disorders [21], and musculoskeletal conditions in older adults [22]. Some studies have investigated exercise programs in individuals with chronic neck pain, focusing on improvements in pain, disability, and quality of life [23,24]. However, the effectiveness of a telerehabilitation-based therapeutic exercise program specifically targeting pain, disability, cervical angle, and ROM in patients with neck pain combined with FHP has not yet been clearly established.

This study aims to investigate the effectiveness of an internet-based telerehabilitation exercise program compared with an in-person exercise program for individuals with chronic nonspecific neck pain and FHP. We hypothesize that telerehabilitation will be equally effective as the in-person program in improving pain, cervical angle, ROM, and functional outcomes.

## Methods

### Study Design

This study used a randomized controlled trial design with a 1:1 allocation ratio to compare 2 groups over 6 weeks with a 2-week follow-up, conducted between January and April 2024. All assessments and in-person interventions were conducted at the Physiotherapy Department Laboratory, Walailak University, Thailand, while the telerehabilitation group received their intervention via Zoom (Zoom Video Communications) platform. The study reporting followed the CONSORT-EHEALTH (Consolidated Standards of Reporting Trials of Electronic and Mobile Health Applications and Online Telehealth) reporting guidelines (the CONSORT-EHEALTH checklist is provided in Checklist 1) [25].

### Participants and Randomization

Females aged 18-25 years were eligible to participate in this study and were recruited through public announcement and voluntary sign-up. Inclusion criteria required participants to have nonspecific neck pain that worsened with active neck movement in at least 1 direction. In addition, participants had experienced neck pain for at least 12 weeks and reported a Visual Analog Scale (VAS) score of 3 to 8 out of 10. FHP was defined as a CVA of less than 50°, as established in a previous study [26]. Furthermore, participants in the telerehabilitation group were required to have access to a smartphone or computer with internet connectivity and basic ability to use videoconferencing applications. Exclusion criteria included a history of shoulder or spine injuries or surgeries, spinal deformities (eg, thoracic hyperkyphosis or scoliosis), neurological deficits, infections, inflammatory arthritis in the cervical spine, regular participation in neck and shoulder exercises, or having undergone physical therapy or other medical treatments for neck pain in the previous 6 months. The eligibility criteria and other trial components remained consistent throughout the study, with no major methodological changes made after the trial began.

An a priori power analysis was conducted using G*Power software, version 3.1.9.7 (by Franz Faul, Universität Kiel, Germany) to determine the required sample size. The calculation was based on an *F* test using the effect size reported in a previous study comparing therapeutic exercises with a control group [27]. A total of 42 participants was determined through the calculations. However, a 15% dropout rate was anticipated. As a result, the final target sample size for this study was 50 participants.

Participants were randomly assigned to either the telerehabilitation or in-person group through a concealed allocation process. At the beginning of the program, an independent researcher, not involved in any assessments, prepared 50 small opaque slips labeled “1” and “2.” Each participant drew 1 slip from a container to determine their group assignment. To ensure allocation concealment, sealed and sequentially numbered opaque containers were used, thereby preserving the integrity of the randomization process and minimizing the risk of selection bias. The outcome assessors were blinded to group assignments throughout the study.

### Outcome Measures

Neck pain, CVA, neck disability level, and cervical range of motion (CROM) were evaluated at baseline, after 4 and 6 weeks of intervention, and at a 2-week follow-up. Evaluations were administered in-person in the physiotherapy department laboratory by an assessor who was blinded to group allocation and not involved in the intervention for either group. No modifications were made to the trial outcomes after the commencement of the trial. The outcomes outlined in the protocol remained consistent throughout the study period.

Neck pain was assessed using the VAS within a 24-hour period. The VAS, widely regarded as one of the most reliable and validated methods for self-reporting pain intensity, uses a 10 cm horizontal line. On this scale, 0 cm indicates “no pain,” while 10 cm represents “worst pain” [28].

CVA was measured using photogrammetry in the sitting position. Markers were placed over the right tragus of the ear and the C7 spinous process. Participants were instructed to sit in their neutral resting position with their arms naturally on their knees [29]. A digital camera was positioned on a tripod at shoulder level, 0.8 m away from the participants. Angles were measured using Kinovea, a 2D motion analysis software. CVA was measured from the intersection between a horizontal line through the spinous process of C7 and a line extending to the tragus of the ear. The angle was measured 3 times to enhance data reliability.

Neck disability level was determined using the Neck Disability Index (NDI), Thai version. This self-reported questionnaire determined the disability index of the cervical spine and its impact on daily activities. The NDI has been validated as an effective evaluative tool for patients with neck pain [30].

CROM was measured using a CROM device (Performance Attainment Associates and MedNet Technologies). Active ROM of the cervical spine was assessed in 6 directions, such as flexion, extension, lateral flexion to the left, lateral flexion to the right, rotation to the left, and rotation to the right. The device has demonstrated high intratester reliability (intraclass correlation coefficient [ICC]=0.87‐0.96) for patients with neck pain [31].

Adherence to the intervention was monitored in both groups using attendance logs and considered as a process outcome. All scheduled sessions were monitored, and participant adherence was defined by session attendance.

### Intervention and Control

Following the initial randomization meeting, all participants received patient education regarding postural correction and guidance on avoiding certain activities, particularly rest break guidelines for mobile electronic device and computer use [32]. Both groups received the same exercise program: a combined stretching and strengthening corrective exercise intervention conducted 3 times per week for 6 weeks. The exercise program was designed by a licensed physiotherapist with 18 years of clinical experience in musculoskeletal rehabilitation, based on a synthesis of exercises previously shown to be effective in managing FHP in the literature [27,29,32-34]. Participants were instructed to stretch each muscle, including the pectoralis, sternocleidomastoid, upper trapezius, and levator scapulae, to the end of its range without inducing pain or discomfort, following standard protocols. Each stretch was held for 30 seconds and repeated twice for each muscle on both the right and left sides, with a 30-second rest period between repetitions and between muscles. Four strengthening corrective exercises were performed: sitting chin tuck, standing W-to-Y, wall slide with shoulder flexion while facing the wall, and wall slide with shoulder abduction and external rotation while positioned with the back against the wall. Each exercise consisted of 10 repetitions per set, with 3 sets completed for each exercise. A 1-minute rest period was provided between sets, and a 2-second rest was allowed between exercises. Each session lasted approximately 40 minutes. The exercise protocol and remote supervision model remained unchanged throughout the trial. Participants in both groups received weekly reminders via the LINE application before each scheduled session. The detailed exercise prescription for the strengthening corrective exercises is provided in Multimedia Appendix 1.

The telerehabilitation group performed the exercise program individually under real-time remote supervision in their home environment via secure, password-protected Zoom links. No sessions were recorded, and the Zoom platform remained stable and unchanged during the study. The researcher monitored movement patterns and provided verbal feedback to correct exercises, ensuring both proper technique and compliance with the prescribed exercise schedule.

The in-person group completed the same program under supervision at a physiotherapy laboratory. Another researcher monitored the movement patterns and provided both verbal instructions and manual correction when necessary. This difference in feedback modality was intentional and designed to reflect real-world remote rehabilitation settings, where tactile feedback is not feasible. Therapists followed a standardized session checklist to ensure consistent feedback and technique correction.

During the 6-week intervention period and 2-week follow-up, all participants were instructed to refrain from engaging in any neck-related exercises or receiving physical therapy or other medical treatments for neck pain.

### Statistical Analysis

Statistical analysis was conducted using SPSS for Windows (version 23, IBM Corp). A *P* value of .05 was considered statistically significant. Descriptive statistics, including the mean, SD, and 95% CI values, were reported. Intervention effects on outcome measures were assessed via repeated-measures analysis of variance with a 2-by-4 design (2 groups and 4 time points). The effect size was indicated by partial eta squared (η²). In the case of significant group-by-time interactions, post hoc pairwise comparisons with Bonferroni correction were used to adjust for multiple comparisons. Intention-to-treat analysis was applied to account for altered results from baseline if data were lost during follow-up. No interim analyses were performed during the trial, and stopping guidelines were not relevant. The trial adhered strictly to the predetermined protocol without any deviations concerning interim analyses or early termination.

### Ethical Considerations

The study protocol was approved by the Human Research Ethics Committee of Walailak University, Thailand (approval number: WUEC-23-335-02), and the trial was registered with the Thai Clinical Trials Registry (registration number: TCTR20240326007). The data were deidentified, and no compensation was provided. Before the study began, all participants were informed about the study’s purpose, procedures, potential risks and benefits, their right to withdraw at any time without consequences, and how their personal data would be protected. Written informed consent was obtained from all participants.

## Results

**Figure 1.**
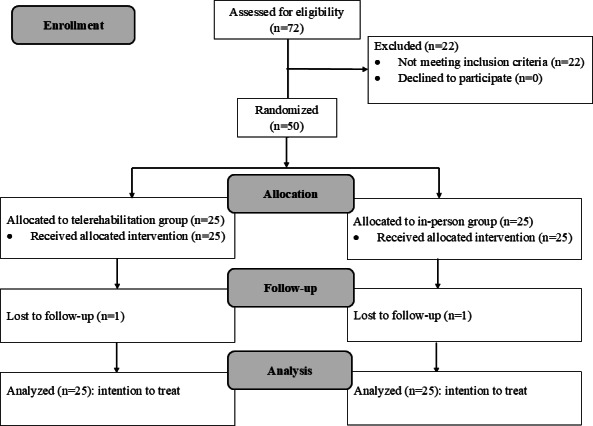
Consolidated Standards of Reporting Trials diagram for the randomized clinical trial.

This study initially recruited 72 participants for screening, of which 22 were disqualified, leaving 50 participants enrolled. A total of 50 participants were randomized into telerehabilitation and in-person groups. A depiction of a participant in the trial is presented in Figure 1.

The participants’ characteristics are presented in Table 1. One participant from the telerehabilitation group and another participant from the in-person group withdrew, leading to a dropout. Among the 48 participants who completed the intervention, adherence was 100% (48/48), with all participants attending every scheduled session over the 6-week period. The intention-to-treat principle was used, and all 50 participants were included in the analysis (Figure 1). The baseline characteristics were similar between the groups for all variables. No harm or unintended effects were observed in either group. The participants reported no adverse events during the treatment period, nor were any identified during the 2-week follow-up. No additional analyses, such as subgroup analyses or adjusted analyses, were conducted.

**Table 1.** Demographic characteristics of participants in the telerehabilitation and in-person groups.

Characteristics	Telerehabilitation (n=25), mean (SD)	In-person (n=25), mean (SD)	*P* value^a^
Age (years)	19.80 (1.29)	19.76 (1.27)	.91
BMI (kg/m^2^)	22.77 (3.91)	23.15 (4.13)	.74
Pain duration (months)	12.20 (9.59)	16.16 (9.40)	.15

a *P* values were calculated using independent *t* tests.

**Figure 2.**
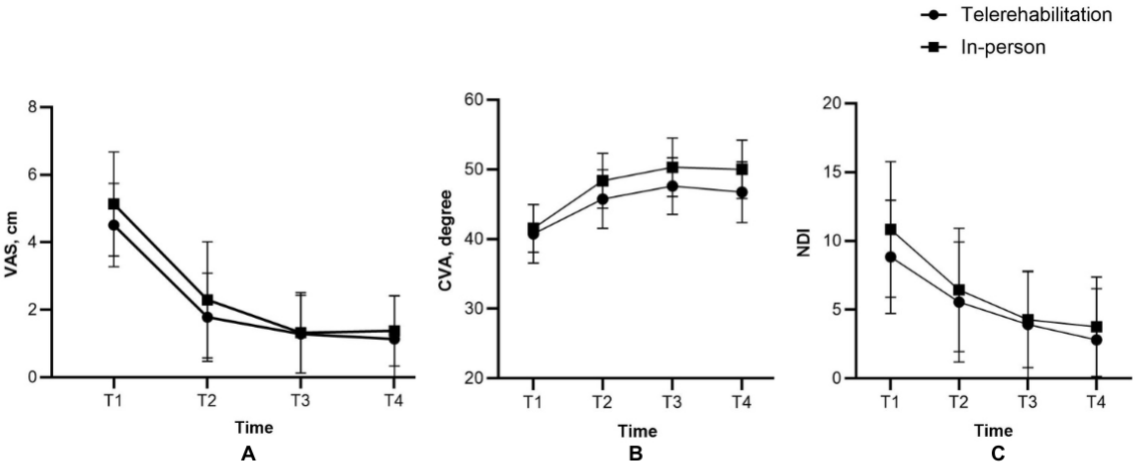
Visual analog scale (A), craniovertebral angle (B), and neck disability index (C) at 4-time points (baseline, after 4-week and 6-week of intervention, and during 2-week of follow-up). T1=week0, T2=4-week treatment, T3=6-week treatment, and T4=2-week follow-up. CVA: craniovertebral angle; NDI: neck disability index; VAS: visual analog scale.

Mean (SD) of VAS, CVA, NDI, and CROM at baseline, after 4 weeks of intervention, after 6 weeks of intervention, and during a 2-week follow-up period of both telerehabilitation and in-person groups were presented in Table 2 and Figures2  3.

Table 3 presents the mean change from baseline with 95% CI values for all outcomes within each group over time. The multivariate test demonstrated that the main effect of group-by-time interaction had no significant difference of all variables including VAS (*F*_3,46_=1.001; *P*=.40; η²=0.061), CVA (*F*_3,46_=1.554; *P*=.21; η²=0.092), NDI (*F*_3,46_=1.489; *P*=.23; η²=0.089), CROM of flexion (*F*_3,46_=0.610; *P*=.61; η²=0.038), CROM of extension (*F*_3,46_=2.181; *P*=.10; η²=0.125), CROM of lateral flexion to left (*F*_3,46_=1.633; *P*=.19; η²=0.096), CROM of lateral flexion to right (*F*_3,46_=2.529; *P*=.07; η²=0.142), CROM of rotation to left (*F*_3,46_=1.102; *P*=.36; η²=0.067), and CROM of rotation to right (*F*_3,46_=1.048; *P*=.38; η²=0.064).

However, the main effect of time was statistically significant in all parameters; VAS (*F*_3,46_=85.673; *P*<.001; η²=0.848), CVA (*F*_3,46_=69.383; *P*<.001; η²=0.819), NDI (*F*_3,46_=49.704; *P*<.001; η²=0.764), CROM of flexion (*F*_3,46_=61.035; *P*<.001; η²=0.799), CROM of extension (*F*_3,46_=35.699; *P*<.001; η²=0.700), CROM of lateral flexion to left (*F*_3,46_=86.050; *P*<.001; η²=0.849), CROM of lateral flexion to right (*F*_3,46_=62.989; *P*<.001; η²=0.804), CROM of rotation to left (*F*_3,46_=57.219; *P*<.001; η²=0.789), and CROM of rotation to right (*F*_3,46_=140.378; *P*<.001; η²=0.902).

Pairwise comparisons using Bonferroni correction revealed statistically significant improvements from baseline (week 0) at all subsequent time points (weeks 4-, 6-, and 2-week follow-up) in both the telerehabilitation and in-person groups. Significant improvements from baseline were observed in all outcomes, including VAS (*P*<.001), CVA (*P*<.001), NDI (*P*<.001), and CROM in flexion, extension, lateral flexion, and rotation (*P*<.001*-P*=.04, depending on direction and group).

**Table 2.** Mean (SD) score by group over time.

Outcome measure	Week 0 (baseline), mean (SD)		4-week treatment, mean (SD)		6-week treatment, mean (SD)		2-week follow-up, mean (SD)	
	^a^TR (n=25)	^b^IP (n=25)	TR (n=25)	IP (n=25)	TR (n=25)	IP (n=25)	TR (n=25)	IP (n=25)
^c^VAS (cm)	4.51 (1.23)	5.14 (1.54)	1.78 (1.30)	2.30 (1.72)	1.28 (1.15)	1.32 (1.19)	1.14 (1.28)	1.38 (1.04)
^d^CVA (°)	40.75 (4.21)	41.54 (3.45)	45.76 (4.22)	48.37 (3.96)	47.63 (4.08)	50.32 (4.19)	46.76 (4.39)	50.02 (4.20)
^e^NDI (score)	8.84 (4.12)	10.84 (4.93)	5.56 (4.36)	6.44 (4.48)	3.92 (3.88)	4.28 (3.49)	2.80 (3.72)	3.76 (3.62)
^f^CROM-Flexion (°)	51.93 (8.29)	51.64 (7.82)	61.49 (9.64)	59.17 (9.30)	65.40 (8.27)	65.07 (6.78)	59.90 (7.75)	62.21 (7.85)
CROM-Extension (°)	54.25 (9.10)	55.48 (12.37)	61.07 (9.74)	58.97 (13.26)	67.47 (6.86)	67.14 (12.22)	63.49 (6.87)	60.91 (12.36)
CROM-Lateral flexion to left (°)	36.13 (3.07)	36.79 (3.36)	40.15 (2.91)	41.07 (2.95)	41.99 (2.10)	43.89 (2.74)	40.61 (2.01)	42.07 (2.65)
CROM-Lateral flexion to right (°)	36.16 (3.05)	36.63 (3.42)	40.37 (2.27)	40.55 (2.61)	41.29 (2.36)	41.81 (2.78)	40.73 (2.39)	42.21 (2.47)
CROM-Rotation to left (°)	55.17 (7.17)	55.29 (7.67)	61.22 (5.93)	63.07 (6.93)	64.51 (5.48)	65.07 (5.05)	62.48 (4.66)	62.59 (5.26)
CROM-Rotation to right (°)	41.14 (6.67)	41.68 (8.31)	58.16 (8.32)	57.55 (9.24)	62.45 (8.03)	63.36 (7.20)	60.75 (9.13)	63.84 (8.19)

a TR: telerehabilitation group.

b IP: in-person group.

c VAS: visual analog scale.

d CVA: craniovertebral angle.

e NDI: neck disability index.

f CROM: cervical range of motion.

**Figure 3.**
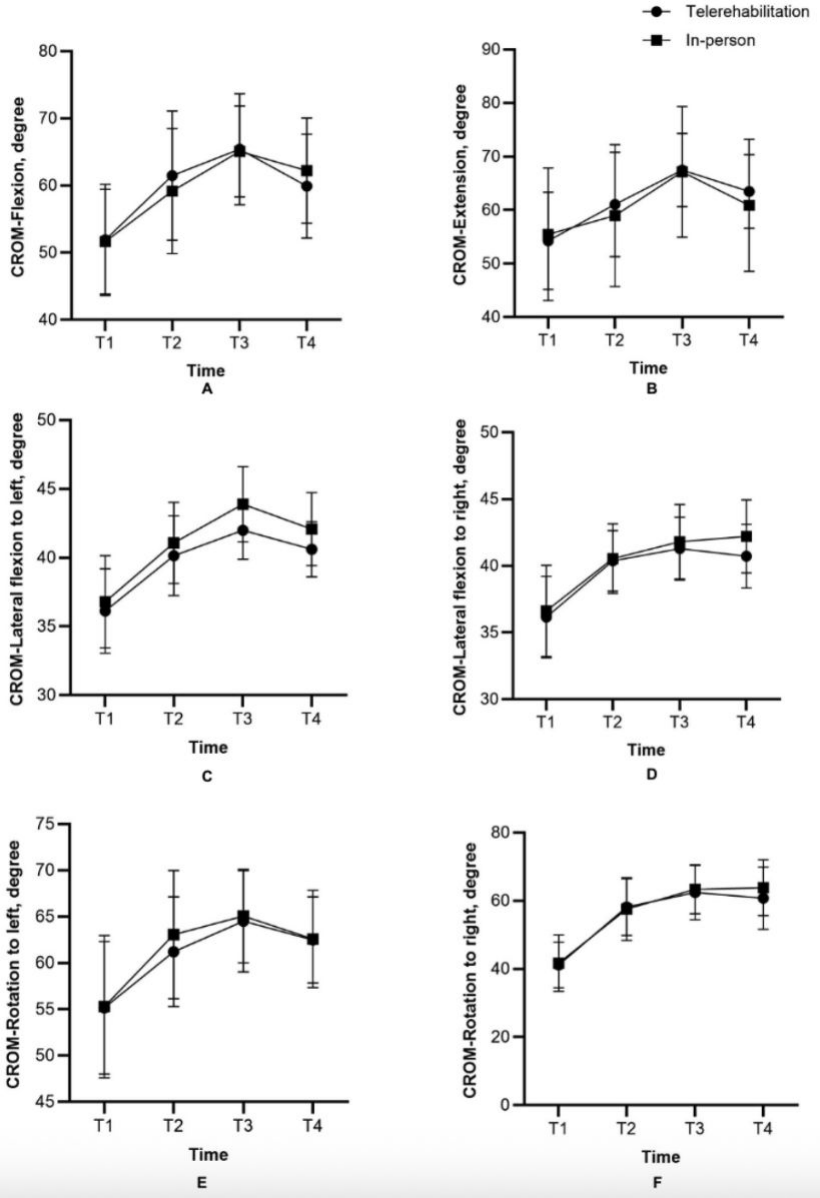
Cervical range of motion: flexion (A), extension (B), lateral flexion to left (C), lateral flexion to right (D), rotation to left (E), and rotation to right (F) at 4-time points (baseline, after 4-week and 6-week of intervention, and during 2-week of follow-up). T1=week0, T2=4-week treatment, T3=6-week treatment, T4=2-week follow-up. CROM: cervical range of motion.

**Table 3.** Mean (95% CI) change within group over time.

Outcome measure	Mean (95% CI) change within group					
	Week 4−week 0		Week 6−week 0		F/U 2 week−week 0	
	TR^a^ (n=25)	IP^b^ (n=25)	TR (n=25)	IP (n=25)	TR (n=25)	IP (n=25)
VAS^c^ (cm)	2.188 (1.26 to 3.11)	2.812 (1.88 to 3.74)	3.104 (2.26 to 3.95)	3.876 (3.03 to 4.72)	3.428 (2.51 to 4.35)	4.140 (3.22 to 5.06)
CVA^d^ (°)	−5.012 (−7.33 to −2.69)	−6.832 (−9.15 to −4.51)	−6.884 (−9.29 to −4.48)	−8.784 (−11.19 to −6.38)	−6.012 (−8.49 to −3.54)	−8.488 (−10.96 to −6.01)
NDI^e^ (score)	3.280 (1.34 to 5.22)	4.40 (2.46 to 6.34)	4.920 (2.93 to 6.91)	6.56 (4.57 to 8.55)	6.04 (3.97 to 8.11)	7.08 (5.01 to 9.15)
CROM-Flexion^f^ (°)	−9.56 (−13.96 to −5.16)	−7.53 (−11.93 to −3.14)	−13.47 (−17.29 to −9.64)	−13.43 (−17.25 to −9.60)	−10.28 (−14.28 to −6.28)	−8.27 (−12.27 to −4.27)
CROM-Extension (°)	−6.81 (−11.23 to −2.40)	−3.49 (−7.91 to 0.92)	−13.23 (−18.07 to −8.38)	−11.67 (−16.51 to −6.82)	−9.24 (−13.26 to −5.22)	−5.43 (−9.15 to −1.41)
CROM- Lateral flexion to left (°)	−4.01 (−5.66 to −2.37)	−4.28 (−5.93 to −2.63)	−5.87 (−5.66 to −2.37)	−7.11 (−8.75 to −5.46)	−4.48 (−6.05 to −2.91)	−5.28 (−6.82 to −3.71)
CROM- Lateral flexion to right (°)	−4.21 (−5.66 to −2.77)	−3.92 (−5.36 to −2.48)	−5.13 (−7.05 to −3.21)	−5.19 (−7.11 to −3.27)	−4.57 (−5.98 to −3.17)	−5.59 (−6.99 to −4.18)
CROM- Rotation to left (°)	−6.05 (−8.29 to −3.82)	−7.77 (−10.01 to −5.54)	−9.33 (−12.94 to −5.73)	−9.77 (−13.38 to −6.17)	−7.31 (−10.42 to −4.19)	−7.29 (−10.41 to −4.18)
CROM- Rotation to right (°)	−17.01 (−21.75 to −12.27)	−15.87 (−20.61 to −11.13)	−21.31 (−25.44 to −17.18)	−21.68 (−25.81 to −17.55)	−22.69 (−27.95 to −17.43)	−19.07 (−24.33 to −13.81)

a TR: telerehabilitation group.

b IP: in-person group.

c VAS: visual analog scale.

d CVA: craniovertebral angle.

e NDI: neck disability index.

f CROM: cervical range of motion.

## Discussion

### Principal Findings

The major finding of this study is that there were no significant differences in neck pain intensity (VAS), CVA, NDI, and CROM between the telerehabilitation and in-person groups, indicating comparable effectiveness of both delivery methods. The partial eta squared values supported the overall interpretation that both telerehabilitation and in-person exercise produced clinically meaningful improvements. The consistently large effect sizes observed for the main effect of time further emphasize the effectiveness of the intervention across both delivery methods. In addition, adherence to the intervention was excellent, with all participants who completed the program attending all scheduled sessions. This high adherence rate demonstrates the feasibility and acceptance of both approaches among participants. These findings support the clinical viability of remote rehabilitation models when implemented with appropriate supervision and technological support.

This exercise program can be effectively delivered via telemedicine using real-time videoconferencing for young adult females with FHP and nonspecific chronic neck pain. The intervention led to improvements in neck pain management, cervical alignment, neck disability, and ROM. Furthermore, physical therapy services delivered through real-time interactive communication provide significant benefits for patients who are geographically isolated, reduce travel distances, and require more timely responses for care [35].

In this study, both the telerehabilitation and in-person groups, which followed an identical exercise protocol, demonstrated comparable improvements in neck pain. This supports the effectiveness of a combined stretching and corrective strengthening program delivered over 4-6 weeks in reducing pain intensity. A previous study reported that neck pain is often associated with poor posture and FHP, both of which are linked to muscle imbalances [36]. Therefore, therapeutic exercises can effectively address potential muscle imbalances [37]. Iqbal et al [38] emphasized the clinical importance of deep cervical flexor training for managing neck pain and improving functional disability. Letafatkar et al [27] revealed that corrective exercises targeting the neck and upper body can effectively reduce neck pain. Furthermore, changes in CVA have been identified as being strongly associated with variations in pain levels [39]. In this study, the difference in VAS from baseline in both groups exceeded the minimum clinically important difference (MCID) of 2.14 cm [40], indicating a clinically significant effect of the therapeutic exercise program, regardless of whether it was delivered through in-person supervision or real-time videoconferencing. In contrast, previous internet-based interventions for chronic neck pain have shown statistically significant improvements in pain; however, the mean change in VAS did not exceed the MCID threshold [41].

To correct the FHP in this study was achieved by increasing the CVA. The exercise program aimed to restore normal muscle balance between opposing muscle groups. Training the deep neck flexor muscles (longus colli and capitis) resulted in a reduction in the mechanosensitivity of specific neural, muscular, and articular structures [42], thereby improving the ability to maintain a neutral cervical position [43]. In addition, increasing the activation of the lower trapezius and serratus anterior muscles, which are critical for scapular stabilization, contributed to reducing abnormal posture [44]. Most studies, including this one, have used a combination of stretching and strengthening exercises with varying characteristics to improve CVA [45,46]. A meta-analysis demonstrated significant improvements in CVA across participants of diverse age ranges and socioeconomic groups when various therapeutic exercises were applied. Specifically, individuals with neck pain experienced a mean improvement in CVA of 4.58° [39]. These findings suggest that both telerehabilitation and in-person exercise programs could enhance CVA outcomes beyond previously reported levels.

Both the telerehabilitation and in-person groups demonstrated a statistically significant improvement in neck-related functional disability, as measured by the NDI. Following a 6-week intervention, the NDI reached the minimum detectable change threshold of at least 5 points but did not surpass the MCID of a 7-point reduction required to confirm meaningful clinical improvement [47]. This suggests that, while the intervention had a measurable effect on disability, the extent of change may not have been large enough for participants to perceive a substantial functional benefit. This limitation should be considered when interpreting the clinical relevance of the NDI findings. Nonetheless, a previous study reported that therapeutic exercises for the neck achieved a faster reduction in neck disability compared with manual therapy [48].

CROM in all directions improved in both groups, potentially due to pain relief. A previous study showed that patients with mechanical neck pain could enhance CROM by improving joint mobility and reducing soft tissue adhesions [49]. Similarly, Anderson et al [50] reported that a 6-week cervical stretching and strengthening exercise program improved neck ROM in young adults, regardless of whether they experienced neck pain.

Telerehabilitation delivered through real-time videoconferencing offers practical benefits for patients familiar with digital tools. In this study, all participants were able to access the Zoom-based exercise sessions without difficulty. Previous research has shown that factors, such as age, cognitive reserve, and therapist support can influence satisfaction and engagement with telerehabilitation [51,52]. Therapists play a key role in guiding patients through technology use and providing motivational support to maximize treatment adherence. One notable difference between groups was the mode of therapist feedback. While the in-person group received occasional manual corrections, the telerehabilitation group relied solely on verbal and visual guidance. Despite this difference, both groups demonstrated comparable improvements, suggesting that effective exercise instruction and correction can be achieved through telerehabilitation without the need for tactile feedback. This supports the clinical use of remote physiotherapy models, especially in settings where physical interaction is limited.

Importantly, our findings demonstrate that telerehabilitation is clinically equivalent to in-person supervision in improving pain, posture, neck disability, and CROM in individuals with chronic neck pain and FHP. This equivalence supports its use as a valid alternative to in-person care. With comparable outcomes in both groups, telerehabilitation may help expand access to effective physiotherapy, especially for individuals living in remote or underserved areas. As digital literacy improves, remote physiotherapy models may become more sustainable in routine clinical practice.

### Limitations and Future Research

This study had some limitations. First, exercises performed in a lying position were excluded because telerehabilitation required participants to remain in front of a mobile device or laptop for the physiotherapist to observe and provide feedback effectively. Second, although corrective strengthening exercises were included, cervical and upper body muscle strength was not assessed. This limits interpretation regarding whether observed improvements were linked to actual strength gains. Including muscle strength assessment would have provided a more comprehensive understanding of treatment effects. Third, participant blinding was not feasible due to the nature of the intervention, a common constraint in exercise studies. However, assessor blinding and concealed allocation were used to minimize bias. Fourth, participant satisfaction was not evaluated, restricting insight into the user experience with telerehabilitation. Finally, the study focused on young adult females, limiting generalizability to other sexes and age groups. These limitations indicate that findings should be applied cautiously to broader populations, particularly males, older adults, or those requiring long-term care.

Furthermore, understanding the cost-effectiveness and satisfaction associated with telerehabilitation is essential for guiding future clinical implementation. Future research should explore the long-term sustainability of treatment effects, cost-effectiveness, and broader applicability of telerehabilitation across diverse populations and clinical settings. To better understand treatment response and optimize intervention strategies, future studies should incorporate subgroup analyses based on baseline characteristics such as pain duration and include objective measures of cervical and upper body muscle strength to more accurately assess the physiological impact of corrective exercises.

### Conclusions

This study demonstrates that real-time supervised telerehabilitation can achieve clinical outcomes comparable to in-person physiotherapy in improving pain, posture, disability, and cervical range of motion among young adult females with chronic neck pain and forward head posture. These findings provide strong support for the use of telerehabilitation as a clinically effective, feasible, and accessible treatment option, particularly in settings where in-person care is not readily available.

## Supplementary material

10.2196/74979 Multimedia Appendix 1 Detailed exercise prescription for strengthening corrective exercises.

10.2196/74979 Checklist 1 CONSORT-EHEALTH checklist (V1.6.1).

## Abbreviation

CONSORT-EHEALTH: Consolidated Standards of Reporting Trials of Electronic and Mobile Health Applications and Online Telehealth
CROM: cervical range of motion
CVA: craniovertebral angle
FHP: forward head posture
MCID: minimum clinically important difference
NDI: Neck Disability Index
ROM: range of motion
VAS: Visual Analog Scale
