# Supplementary material for: Internet-Based Telerehabilitation Versus in-Person Therapeutic Exercises in Young Adult Females With Chronic Neck Pain and Forward Head Posture: Randomized Controlled Trial
Source: JMIR Rehabil Assist Technol. 2025 Jul 25;12:e74979. doi: 10.2196/74979 (PMC12296222; doi:10.2196/74979)
Supplement: Multimedia Appendix 1 [file rehab-v12-e74979-s001.docx]

**Multimedia Appendix 1**: Detailed Exercise Prescription for Strengthening Corrective Exercises

| **Strengthening corrective exercise** | **Exercise prescription** | **Pictures** |
| --- | --- | --- |
| Chin tuck in sitting | The participant sits upright on a chair with feet flat on the ground, maintaining an erect posture with a straight back and relaxed shoulders. The chin is gently retracted backward to create a “double chin”, ensuring that the head moves directly backward without tilting upward or downward. A fingertip placed on the chin may be used to guide the movement. The neck and shoulders remain relaxed throughout the exercise. The participant then slowly returns to the starting position. | 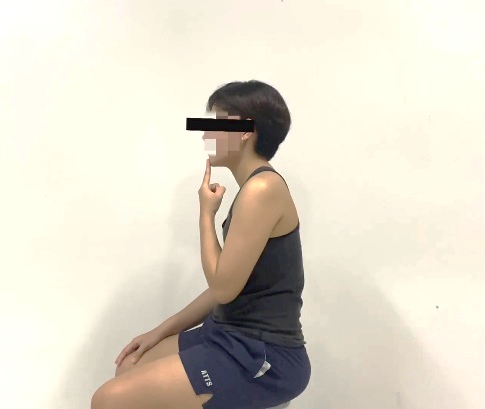  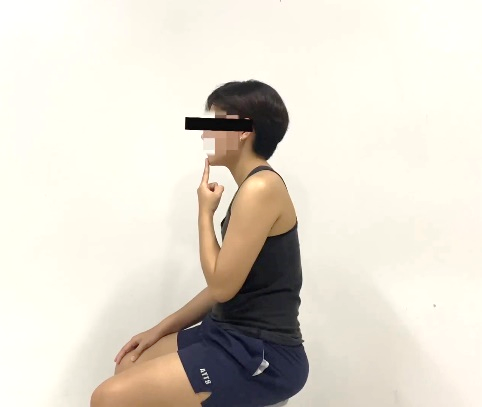 |
| Standing W to Y | The participant positions their arms to form the letter “W” by abducting the shoulders to 90° and flexing the elbows to 90°. The scapulae are retracted, and the arms are externally rotated while maintaining 90° of shoulder abduction. Subsequently, the participant transitions to forming the letter “Y” by raising the arms overhead while maintaining scapular retraction. The elbows are fully extended as the arms and body align to form the letter “Y”. | 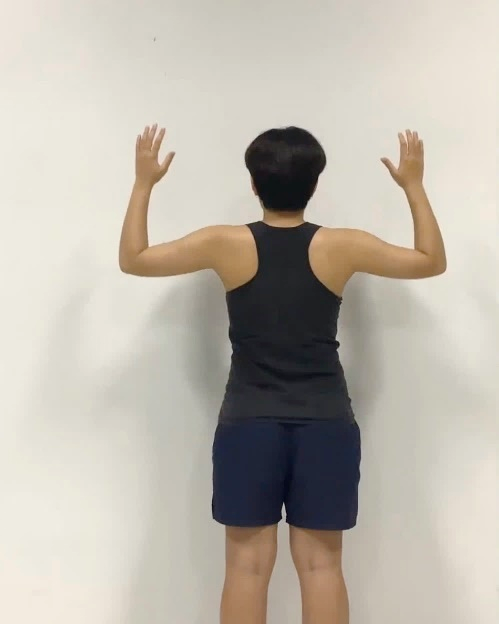  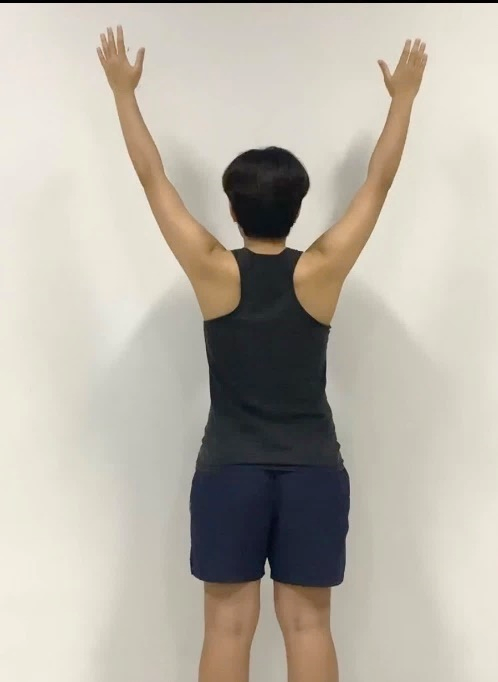 |
| Wall slide with shoulder flexion while facing the wall | The participant faces the wall and places the ulnar sides of their hands against the wall with the shoulders in a flexed position. While gently tucking the chin toward the front of the neck, the participant slides their arms upward along the wall, maintaining the cervical spine in a neutral position and avoiding compensatory cervical extension during shoulder flexion. The arms are then returned to the starting position. | 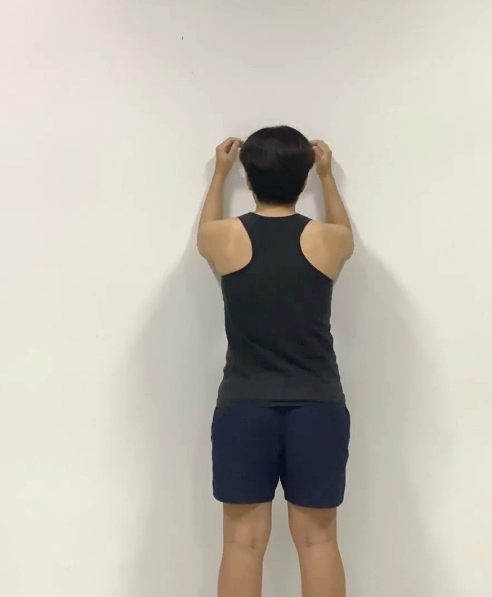  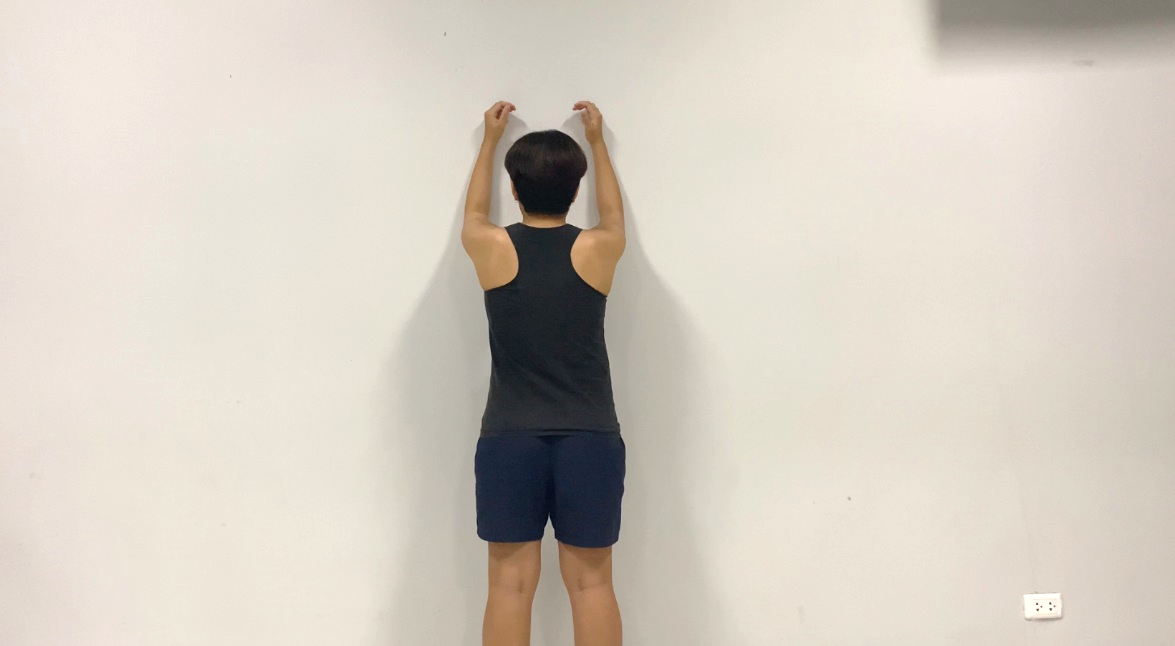 |
| Wall slide with shoulder abduction and external rotation while positioned with the back against the wall | The participant stands with their back against the wall. The shoulder joint is positioned in 90° of abduction and external rotation, while the elbow joint is flexed at 90°. The arms are then elevated and lowered, engaging the lower trapezius muscles while minimizing compensation by the upper trapezius. | 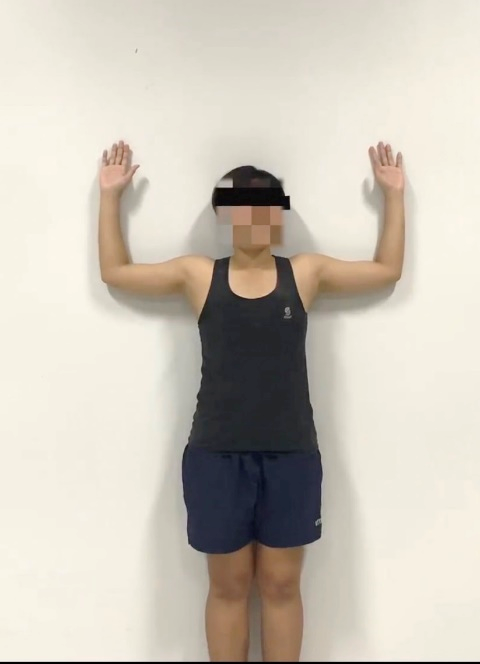  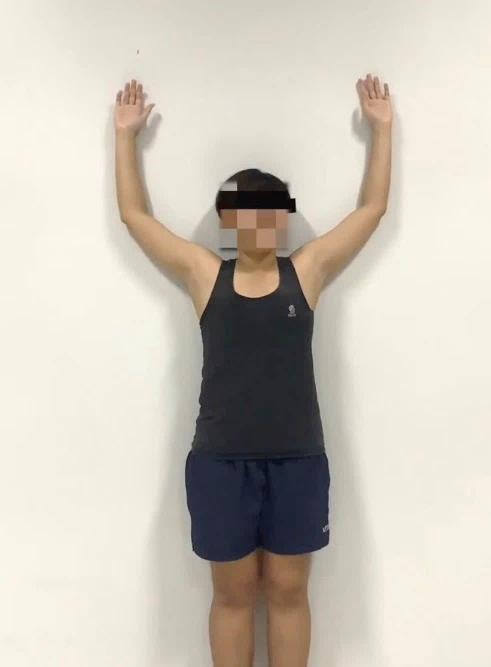 |
