# Supplementary material for: Internet-Based Telerehabilitation Versus in-Person Therapeutic Exercises in Young Adult Females With Chronic Neck Pain and Forward Head Posture: Randomized Controlled Trial
Source: JMIR Rehabil Assist Technol. 2025 Jul 25;12:e74979. doi: 10.2196/74979 (PMC12296222; doi:10.2196/74979)
Supplement: Checklist 1 [file rehab-v12-e74979-s002.pdf]

CONSORT-EHEALTH (V 1.6.1) - Submission/Publication Form

The CONSORT-EHEALTH checklist is intended for authors of randomized trials evaluating web-based and Internet-based applications/interventions, including mobile interventions, electronic games (incl multiplayer games), social media, certain telehealth applications, and other interactive and/or networked electronic applications. Some of the items (e.g. all subitems under item 5 - description of the intervention) may also be applicable for other study designs.

The goal of the CONSORT EHEALTH checklist and guideline is to be
a) a guide for reporting for authors of RCTs,
b) to form a basis for appraisal of an ehealth trial (in terms of validity)

CONSORT-EHEALTH items/subitems are MANDATORY reporting items for studies published in the Journal of Medical Internet Research and other journals / scientific societies endorsing the checklist.

Items numbered 1., 2., 3., 4a., 4b etc are original CONSORT or CONSORT-NPT (non-pharmacologic treatment) items.
Items with Roman numerals (i., ii, iii, iv etc.) are CONSORT-EHEALTH extensions/clarifications.

As the CONSORT-EHEALTH checklist is still considered in a formative stage, we would ask that you also RATE ON A SCALE OF 1-5 how important/useful you feel each item is FOR THE PURPOSE OF THE CHECKLIST and reporting guideline (optional).

Mandatory reporting items are marked with a red *.
In the textboxes, either copy & paste the relevant sections from your manuscript into this form - please include any quotes from your manuscript in QUOTATION MARKS,
or answer directly by providing additional information not in the manuscript, or elaborating on why the item was not relevant for this study.

YOUR ANSWERS WILL BE PUBLISHED AS A SUPPLEMENTARY FILE TO YOUR PUBLICATION IN JMIR AND ARE CONSIDERED PART OF YOUR PUBLICATION (IF ACCEPTED).
Please fill in these questions diligently. Information will not be copyedited, so please use proper spelling and grammar, use correct capitalization, and avoid abbreviations.

DO NOT FORGET TO SAVE AS PDF _AND_ CLICK THE SUBMIT BUTTON SO YOUR ANSWERS ARE IN OUR DATABASE !!!

Citation Suggestion (if you append the pdf as Appendix we suggest to cite this paper in the caption):
Eysenbach G, CONSORT-EHEALTH Group
CONSORT-EHEALTH: Improving and Standardizing Evaluation Reports of Web-based and Mobile Health Interventions
J Med Internet Res 2011;13(4):e126

URL: http://www.jmir.org/2011/4/e126/
doi: 10.2196/jmir.1923
PMID: 22209829

patcharin.nilmart@gmail.com สลับบัญชี
ไม่ใช้ร่วมกัน
บันทึกฉบับร่างแล้ว

* ระบุว่าเป็นคำถามที่จำเป็น

Your name *
First Last

Patcharin Nilmart

Primary Affiliation (short), City, Country *
University of Toronto, Toronto, Canada

Srinakharinwirot University, Nakhon Nayok, Tha

Your e-mail address *
abc@gmail.com

patcharinn@g.swu.ac.th

Title of your manuscript *
Provide the (draft) title of your manuscript.

Internet-based Telerehabilitation vs In-person Therapeutic Exercises in Young Adult Females with Chronic Neck Pain and Forward Head Posture: Randomized Controlled Trial

Name of your App/Software/Intervention *

If there is a short and a long/alternate name, write the short name first and add the long name in brackets.

Telerehabilitation for neck pain and forward he

Evaluated Version (if any)

e.g. "V1", "Release 2017-03-01", "Version 2.0.27913"

Version 2.0

Language(s) *

What language is the intervention/app in? If multiple languages are available, separate by comma (e.g. "English, French")

English

URL of your Intervention Website or App

e.g. a direct link to the mobile app on app in appstore (itunes, Google Play), or URL of the website. If the intervention is a DVD or hardware, you can also link to an Amazon page.

คำตอบของคุณ

URL of an image/screenshot (optional)

คำตอบของคุณ

Accessibility *

Can an enduser access the intervention presently?

- ◯ access is free and open
- ◯ access only for special usergroups, not open
- ◯ access is open to everyone, but requires payment/subscription/in-app purchases
- ◉ app/intervention no longer accessible
- ◯ อื่นๆ:

Primary Medical Indication/Disease/Condition *

e.g. "Stress", "Diabetes", or define the target group in brackets after the condition, e.g. "Autism (Parents of children with)", "Alzheimers (Informal Caregivers of)"

Young adult females with chronic non-specific

Primary Outcomes measured in trial *

comma-separated list of primary outcomes reported in the trial

Neck pain, Craniovertebral angle

Secondary/other outcomes

Are there any other outcomes the intervention is expected to affect?

neck disability level, cervical range of motion (CROM)

Recommended "Dose" *

What do the instructions for users say on how often the app should be used?

- ○ Approximately Daily
- ○ Approximately Weekly
- ○ Approximately Monthly
- ○ Approximately Yearly
- ○ "as needed"
- ◉ อื่นๆ: three times a week for six weeks

Approx. Percentage of Users (starters) still using the app as recommended after 3 months *

- ◉ unknown / not evaluated
- ○ 0-10%
- ○ 11-20%
- ○ 21-30%
- ○ 31-40%
- ○ 41-50%
- ○ 51-60%
- ○ 61-70%
- ○ 71%-80%
- ○ 81-90%
- ○ 91-100%
- ○ อื่นๆ:

Overall, was the app/intervention effective? *

- ○ yes: all primary outcomes were significantly better in intervention group vs control
- ○ partly: SOME primary outcomes were significantly better in intervention group vs control
- ○ no statistically significant difference between control and intervention
- ○ potentially harmful: control was significantly better than intervention in one or more outcomes
- ○ inconclusive: more research is needed
- ◉ อื่นๆ: Both telerehabilitation and in-person supervision were similarly effectiv

Article Preparation Status/Stage *

At which stage in your article preparation are you currently (at the time you fill in this form)

- ○ not submitted yet - in early draft status
- ○ not submitted yet - in late draft status, just before submission
- ○ submitted to a journal but not reviewed yet
- ◉ submitted to a journal and after receiving initial reviewer comments
- ○ submitted to a journal and accepted, but not published yet
- ○ published
- ○ อื่นๆ:

Journal *

If you already know where you will submit this paper (or if it is already submitted), please provide the journal name (if it is not JMIR, provide the journal name under "other")

- ○ not submitted yet / unclear where I will submit this
- ○ Journal of Medical Internet Research (JMIR)
- ○ JMIR mHealth and UHealth
- ○ JMIR Serious Games
- ○ JMIR Mental Health
- ○ JMIR Public Health
- ○ JMIR Formative Research
- ◉ Other JMIR sister journal
- ○ อื่นๆ:

Is this a full powered effectiveness trial or a pilot/feasibility trial? *

- ○ Pilot/feasibility
- ◉ Fully powered

Manuscript tracking number *

If this is a JMIR submission, please provide the manuscript tracking number under "other" (The ms tracking number can be found in the submission acknowledgement email, or when you login as author in JMIR. If the paper is already published in JMIR, then the ms tracking number is the four-digit number at the end of the DOI, to be found at the bottom of each published article in JMIR)

- ○ no ms number (yet) / not (yet) submitted to / published in JMIR
- ◉ อื่นๆ: JRAT ms#74979

TITLE AND ABSTRACT

1a) TITLE: Identification as a randomized trial in the title

1a) Does your paper address CONSORT item 1a? *

I.e does the title contain the phrase "Randomized Controlled Trial"? (if not, explain the reason under "other")

- (●) yes
- ( ) อื่นๆ:

1a-i) Identify the mode of delivery in the title

Identify the mode of delivery. Preferably use “web-based” and/or “mobile” and/or “electronic game” in the title. Avoid ambiguous terms like “online”, “virtual”, “interactive”. Use “Internet-based” only if Intervention includes non-web-based Internet components (e.g. email), use “computer-based” or “electronic” only if offline products are used. Use “virtual” only in the context of “virtual reality” (3-D worlds). Use “online” only in the context of “online support groups”. Complement or substitute product names with broader terms for the class of products (such as “mobile” or “smart phone” instead of “iphone”), especially if the application runs on different platforms.

	1	2	3	4	5	
subitem not at all important	( )	( )	( )	( )	(●)	essential

ล้างสิ่งที่เลือก

Does your paper address subitem 1a-i? *

Copy and paste relevant sections from manuscript title (include quotes in quotation marks "like this" to indicate direct quotes from your manuscript), or elaborate on this item by providing additional information not in the ms, or briefly explain why the item is not applicable/relevant for your study

"Internet-based Telerehabilitation"

1a-ii) Non-web-based components or important co-interventions in title

Mention non-web-based components or important co-interventions in title, if any (e.g., “with telephone support”).

	1	2	3	4	5	
subitem not at all important	○	○	◉	○	○	essential

ล้างสิ่งที่เลือก

Does your paper address subitem 1a-ii?

Copy and paste relevant sections from manuscript title (include quotes in quotation marks "like this" to indicate direct quotes from your manuscript), or elaborate on this item by providing additional information not in the ms, or briefly explain why the item is not applicable/relevant for your study

There are no important co-interventions.

1a-iii) Primary condition or target group in the title

Mention primary condition or target group in the title, if any (e.g., “for children with Type I Diabetes”) Example: A Web-based and Mobile Intervention with Telephone Support for Children with Type I Diabetes: Randomized Controlled Trial

	1	2	3	4	5	
subitem not at all important	○	○	○	○	◉	essential

ล้างสิ่งที่เลือก

Does your paper address subitem 1a-iii? *

Copy and paste relevant sections from manuscript title (include quotes in quotation marks "like this" to indicate direct quotes from your manuscript), or elaborate on this item by providing additional information not in the ms, or briefly explain why the item is not applicable/relevant for your study

"Young Adult Females with Chronic Neck Pain and Forward Head Posture"

1b) ABSTRACT: Structured summary of trial design, methods, results, and conclusions

NPT extension: Description of experimental treatment, comparator, care providers, centers, and blinding status.

1b-i) Key features/functionalities/components of the intervention and comparator in the METHODS section of the ABSTRACT

Mention key features/functionalities/components of the intervention and comparator in the abstract. If possible, also mention theories and principles used for designing the site. Keep in mind the needs of systematic reviewers and indexers by including important synonyms. (Note: Only report in the abstract what the main paper is reporting. If this information is missing from the main body of text, consider adding it)

	1	2	3	4	5	
subitem not at all important	○	○	○	○	◉	essential

ล้างสิ่งที่เลือก

Does your paper address subitem 1b-i? *

Copy and paste relevant sections from the manuscript abstract (include quotes in quotation marks "like this" to indicate direct quotes from your manuscript), or elaborate on this item by providing additional information not in the ms, or briefly explain why the item is not applicable/relevant for your study

"Participants were randomly assigned to either a telerehabilitation group or an in-person supervision group. Both groups completed the same six-week, physiotherapist-supervised therapeutic exercise program, delivered via Zoom or in the physiotherapy department laboratory, respectively."

1b-ii) Level of human involvement in the METHODS section of the ABSTRACT

Clarify the level of human involvement in the abstract, e.g., use phrases like “fully automated” vs. “therapist/nurse/care provider/physician-assisted” (mention number and expertise of providers involved, if any). (Note: Only report in the abstract what the main paper is reporting. If this information is missing from the main body of text, consider adding it)

	1	2	3	4	5	
subitem not at all important	○	○	○	○	●	essential

ล้างสิ่งที่เลือก

Does your paper address subitem 1b-ii?

Copy and paste relevant sections from the manuscript abstract (include quotes in quotation marks "like this" to indicate direct quotes from your manuscript), or elaborate on this item by providing additional information not in the ms, or briefly explain why the item is not applicable/relevant for your study

"Both groups completed the same six-week physiotherapist-supervised therapeutic exercise program."

1b-iii) Open vs. closed, web-based (self-assessment) vs. face-to-face assessments in the METHODS section of the ABSTRACT

Mention how participants were recruited (online vs. offline), e.g., from an open access website or from a clinic or a closed online user group (closed usergroup trial), and clarify if this was a purely web-based trial, or there were face-to-face components (as part of the intervention or for assessment). Clearly say if outcomes were self-assessed through questionnaires (as common in web-based trials). Note: In traditional offline trials, an open trial (open-label trial) is a type of clinical trial in which both the researchers and participants know which treatment is being administered. To avoid confusion, use “blinded” or “unblinded” to indicated the level of blinding instead of “open”, as “open” in web-based trials usually refers to “open access” (i.e. participants can self-enrol). (Note: Only report in the abstract what the main paper is reporting. If this information is missing from the main body of text, consider adding it)

	1	2	3	4	5	
subitem not at all important	○	○	○	○	◉	essential

ล้างสิ่งที่เลือก

Does your paper address subitem 1b-iii?

Copy and paste relevant sections from the manuscript abstract (include quotes in quotation marks "like this" to indicate direct quotes from your manuscript), or elaborate on this item by providing additional information not in the ms, or briefly explain why the item is not applicable/relevant for your study

"A randomized controlled trial was conducted with 50 participants experiencing chronic neck pain and FHP, recruited through public announcement and voluntary sign-up." "Both groups completed the same six-week, physiotherapist-supervised therapeutic exercise program, delivered via Zoom or in the physiotherapy department laboratory, respectively."

1b-iv) RESULTS section in abstract must contain use data

Report number of participants enrolled/assessed in each group, the use/uptake of the intervention (e.g., attrition/adherence metrics, use over time, number of logins etc.), in addition to primary/secondary outcomes. (Note: Only report in the abstract what the main paper is reporting. If this information is missing from the main body of text, consider adding it)

	1	2	3	4	5	
subitem not at all important	○	○	○	○	◉	essential

ล้างสิ่งที่เลือก

Does your paper address subitem 1b-iv?

Copy and paste relevant sections from the manuscript abstract (include quotes in quotation marks "like this" to indicate direct quotes from your manuscript), or elaborate on this item by providing additional information not in the ms, or briefly explain why the item is not applicable/relevant for your study

"Of the 50 participants, 48 completed the intervention with one dropout from each group. Adherence among completers was 100 percent in both groups. All 50 participants were included in the analysis using the intention-to-treat principle. "

1b-v) CONCLUSIONS/DISCUSSION in abstract for negative trials

Conclusions/Discussions in abstract for negative trials: Discuss the primary outcome - if the trial is negative (primary outcome not changed), and the intervention was not used, discuss whether negative results are attributable to lack of uptake and discuss reasons. (Note: Only report in the abstract what the main paper is reporting. If this information is missing from the main body of text, consider adding it)

	1	2	3	4	5	
subitem not at all important	○	○	○	○	◉	essential

ล้างสิ่งที่เลือก

Does your paper address subitem 1b-v?

Copy and paste relevant sections from the manuscript abstract (include quotes in quotation marks "like this" to indicate direct quotes from your manuscript), or elaborate on this item by providing additional information not in the ms, or briefly explain why the item is not applicable/relevant for your study

"Both telerehabilitation and in-person supervision were similarly effective in improving pain, posture, neck disability, and cervical range of motion in young adult females with chronic neck pain and FHP. These findings suggest that telerehabilitation may be a feasible and accessible alternative to conventional in-person therapeutic exercise programs for managing chronic neck pain."

INTRODUCTION

2a) In INTRODUCTION: Scientific background and explanation of rationale

2a-i) Problem and the type of system/solution

Describe the problem and the type of system/solution that is object of the study: intended as stand-alone intervention vs. incorporated in broader health care program? Intended for a particular patient population? Goals of the intervention, e.g., being more cost-effective to other interventions, replace or complement other solutions? (Note: Details about the intervention are provided in “Methods” under 5)

	1	2	3	4	5	
subitem not at all important	○	○	○	○	◉	essential

ล้างสิ่งที่เลือก

Does your paper address subitem 2a-i? *

Copy and paste relevant sections from the manuscript (include quotes in quotation marks "like this" to indicate direct quotes from your manuscript), or elaborate on this item by providing additional information not in the ms, or briefly explain why the item is not applicable/relevant for your study

"Neck pain is one of the most common musculoskeletal disorders and represents a significant public health concern within the general population."

"According to recent data, the effectiveness of telerehabilitation, especially for physical therapists, may influence clinical decision making and improve patient outcomes during and after the pandemic [17]. Previous research has demonstrated the effectiveness of telerehabilitation for various musculoskeletal conditions, such as patellofemoral pain [18], knee osteoarthritis [19], low back pain [20], musculoskeletal shoulder disorders [21], and musculoskeletal conditions in older adults [22]. Some studies have investigated exercise programs in individuals with chronic neck pain, focusing on improvements in pain, disability, and quality of life [23, 24]. However, the effectiveness of a telerehabilitation-based therapeutic exercise program specifically targeting pain, disability, cervical angle, and ROM in patients with neck pain combined with FHP has not yet been clearly established."

2a-ii) Scientific background, rationale: What is known about the (type of) system

Scientific background, rationale: What is known about the (type of) system that is the object of the study (be sure to discuss the use of similar systems for other conditions/diagnoses, if appropiate), motivation for the study, i.e. what are the reasons for and what is the context for this specific study, from which stakeholder viewpoint is the study performed, potential impact of findings [2]. Briefly justify the choice of the comparator.

	1	2	3	4	5	
subitem not at all important	○	○	○	○	◉	essential

ล้างสิ่งที่เลือก

Does your paper address subitem 2a-ii? *

Copy and paste relevant sections from the manuscript (include quotes in quotation marks "like this" to indicate direct quotes from your manuscript), or elaborate on this item by providing additional information not in the ms, or briefly explain why the item is not applicable/relevant for your study

"Several studies have reported significant improvements in pain, CVA, neck range of motion (ROM), and function in patients with FHP following in-person exercise interventions [14-16]. The usefulness of telemedicine and telerehabilitation in meeting health care demands during emergencies has become more widely acknowledged by medical professionals since the COVID-19 pandemic. "

"Previous research has demonstrated the effectiveness of telerehabilitation for various musculoskeletal conditions, such as patellofemoral pain [18], knee osteoarthritis [19], low back pain [20], musculoskeletal shoulder disorders [21], and musculoskeletal conditions in older adults [22]. Some studies have investigated exercise programs in individuals with chronic neck pain, focusing on improvements in pain, disability, and quality of life [23, 24]. "

2b) In INTRODUCTION: Specific objectives or hypotheses

Does your paper address CONSORT subitem 2b? *

Copy and paste relevant sections from the manuscript (include quotes in quotation marks "like this" to indicate direct quotes from your manuscript), or elaborate on this item by providing additional information not in the ms, or briefly explain why the item is not applicable/relevant for your study

"This study aims to investigate the effectiveness of an internet-based telerehabilitation exercise program compared to an in-person exercise program for individuals with chronic non-specific neck pain and FHP. We hypothesize that telerehabilitation will be equally effective as the in-person program in improving pain, ROM, and functional outcomes."

METHODS

3a) Description of trial design (such as parallel, factorial) including allocation ratio

Does your paper address CONSORT subitem 3a? *

Copy and paste relevant sections from the manuscript (include quotes in quotation marks "like this" to indicate direct quotes from your manuscript), or elaborate on this item by providing additional information not in the ms, or briefly explain why the item is not applicable/relevant for your study

"This study used a randomized controlled trial design with a 1:1 allocation ratio to compare two groups over six weeks with a two-week follow-up, conducted between January and April 2024."

3b) Important changes to methods after trial commencement (such as eligibility criteria), with reasons

Does your paper address CONSORT subitem 3b? *

Copy and paste relevant sections from the manuscript (include quotes in quotation marks "like this" to indicate direct quotes from your manuscript), or elaborate on this item by providing additional information not in the ms, or briefly explain why the item is not applicable/relevant for your study

"The eligibility criteria and other trial components remained consistent throughout the study, with no major methodological changes made after the trial began."

3b-i) Bug fixes, Downtimes, Content Changes

Bug fixes, Downtimes, Content Changes: ehealth systems are often dynamic systems. A description of changes to methods therefore also includes important changes made on the intervention or comparator during the trial (e.g., major bug fixes or changes in the functionality or content) (5-iii) and other “unexpected events” that may have influenced study design such as staff changes, system failures/downtimes, etc. [2].

	1	2	3	4	5	
subitem not at all important	○	○	●	○	○	essential

ล้างสิ่งที่เลือก

Does your paper address subitem 3b-i?

Copy and paste relevant sections from the manuscript (include quotes in quotation marks "like this" to indicate direct quotes from your manuscript), or elaborate on this item by providing additional information not in the ms, or briefly explain why the item is not applicable/relevant for your study

"During the trial period, no major changes were made to the core functionalities or content of the intervention system. However, minor bug fixes were implemented to improve user interface responsiveness and ensure stable performance. There were no significant downtimes reported. No unexpected events such as staff changes or system failures occurred that would have impacted the study design or data collection process."

4a) Eligibility criteria for participants

Does your paper address CONSORT subitem 4a? *

Copy and paste relevant sections from the manuscript (include quotes in quotation marks "like this" to indicate direct quotes from your manuscript), or elaborate on this item by providing additional information not in the ms, or briefly explain why the item is not applicable/relevant for your study

"Females aged 18 to 25 were eligible to participate in this study. Inclusion criteria required participants to have non-specific neck pain that worsened with active neck movement in at least one direction. In addition, participants have experienced neck pain for at least 12 weeks and reported a Visual Analog Scale (VAS) score of three to eight out of 10. Forward head posture (FHP) was defined as a craniovertebral angle (CVA) of less than 50 degrees, as established in a previous study [26]. Furthermore, participants in the telerehabilitation group were required to have access to a smartphone or computer with internet connectivity and basic ability to use videoconferencing applications. Exclusion criteria included a history of shoulder or spine injuries or surgeries, spinal deformities (e.g., thoracic hyperkyphosis or scoliosis), neurological deficits, infections, inflammatory arthritis in the cervical spine, regular participation in neck and shoulder exercises, or having undergone physical therapy or other medical treatments for neck pain in the previous six months."

4a-i) Computer / Internet literacy

Computer / Internet literacy is often an implicit “de facto” eligibility criterion - this should be explicitly clarified.

	1	2	3	4	5	
subitem not at all important	○	○	○	○	◉	essential

ล้างสิ่งที่เลือก

Does your paper address subitem 4a-i?

Copy and paste relevant sections from the manuscript (include quotes in quotation marks "like this" to indicate direct quotes from your manuscript), or elaborate on this item by providing additional information not in the ms, or briefly explain why the item is not applicable/relevant for your study

"participants in the telerehabilitation group were required to have access to a smartphone or computer with internet connectivity and basic ability to use videoconferencing applications."

4a-ii) Open vs. closed, web-based vs. face-to-face assessments:

Open vs. closed, web-based vs. face-to-face assessments: Mention how participants were recruited (online vs. offline), e.g., from an open access website or from a clinic, and clarify if this was a purely web-based trial, or there were face-to-face components (as part of the intervention or for assessment), i.e., to what degree got the study team to know the participant. In online-only trials, clarify if participants were quasi-anonymous and whether having multiple identities was possible or whether technical or logistical measures (e.g., cookies, email confirmation, phone calls) were used to detect/prevent these.

	1	2	3	4	5	
subitem not at all important	○	○	○	○	◉	essential

ล้างสิ่งที่เลือก

Does your paper address subitem 4a-ii? *

Copy and paste relevant sections from the manuscript (include quotes in quotation marks "like this" to indicate direct quotes from your manuscript), or elaborate on this item by providing additional information not in the ms, or briefly explain why the item is not applicable/relevant for your study

"Females aged 18 to 25 were eligible to participate in this study and were recruited through public announcement and voluntary sign-up."
"Evaluations were administered in-person in the physiotherapy department laboratory by an assessor who was blinded to group allocation and not involved in the intervention for either group. "

4a-iii) Information giving during recruitment

Information given during recruitment. Specify how participants were briefed for recruitment and in the informed consent procedures (e.g., publish the informed consent documentation as appendix, see also item X26), as this information may have an effect on user self-selection, user expectation and may also bias results.

	1	2	3	4	5	
subitem not at all important	○	○	○	○	◉	essential

ล้างสิ่งที่เลือก

Does your paper address subitem 4a-iii?

Copy and paste relevant sections from the manuscript (include quotes in quotation marks "like this" to indicate direct quotes from your manuscript), or elaborate on this item by providing additional information not in the ms, or briefly explain why the item is not applicable/relevant for your study

"Before the study began, all participants were informed about the study’s purpose, procedures, potential risks and benefits, their right to withdraw at any time without consequences, and how their personal data would be protected. Written informed consent was obtained from all participants. "

4b) Settings and locations where the data were collected

Does your paper address CONSORT subitem 4b? *

Copy and paste relevant sections from the manuscript (include quotes in quotation marks "like this" to indicate direct quotes from your manuscript), or elaborate on this item by providing additional information not in the ms, or briefly explain why the item is not applicable/relevant for your study

"All assessments and in-person interventions were conducted at the Physiotherapy Department Laboratory, Walailak University, Thailand, while the telerehabilitation group received their intervention online. "

4b-i) Report if outcomes were (self-)assessed through online questionnaires

Clearly report if outcomes were (self-)assessed through online questionnaires (as common in web-based trials) or otherwise.

	1	2	3	4	5	
subitem not at all important	○	○	○	○	◉	essential

ล้างสิ่งที่เลือก

Does your paper address subitem 4b-i? *

Copy and paste relevant sections from the manuscript (include quotes in quotation marks "like this" to indicate direct quotes from your manuscript), or elaborate on this item by providing additional information not in the ms, or briefly explain why the item is not applicable/relevant for your study

Outcomes in this study were not assessed through questionnaires. All assessments were conducted in-person by trained evaluators using standardized clinical tools.

4b-ii) Report how institutional affiliations are displayed

Report how institutional affiliations are displayed to potential participants [on ehealth media], as affiliations with prestigious hospitals or universities may affect volunteer rates, use, and reactions with regards to an intervention.(Not a required item – describe only if this may bias results)

	1	2	3	4	5	
subitem not at all important	○	○	◉	○	○	essential

ล้างสิ่งที่เลือก

Does your paper address subitem 4b-ii?

Copy and paste relevant sections from the manuscript (include quotes in quotation marks "like this" to indicate direct quotes from your manuscript), or elaborate on this item by providing additional information not in the ms, or briefly explain why the item is not applicable/relevant for your study

Institutional affiliations were not prominently displayed in the intervention materials, and are unlikely to have influenced participant behavior.

5) The interventions for each group with sufficient details to allow replication, including how and when they were actually administered

5-i) Mention names, credential, affiliations of the developers, sponsors, and owners

Mention names, credential, affiliations of the developers, sponsors, and owners [6] (if authors/evaluators are owners or developer of the software, this needs to be declared in a “Conflict of interest” section or mentioned elsewhere in the manuscript).

	1	2	3	4	5	
subitem not at all important	○	○	○	○	◉	essential

ล้างสิ่งที่เลือก

Does your paper address subitem 5-i?

Copy and paste relevant sections from the manuscript (include quotes in quotation marks "like this" to indicate direct quotes from your manuscript), or elaborate on this item by providing additional information not in the ms, or briefly explain why the item is not applicable/relevant for your study

The manuscript does not specify the name, credentials, or institutional affiliation of the developer of the exercise program. "The exercise program was designed by a licensed physiotherapist with 18 years of clinical experience of musculoskeletal rehabilitation, based on a synthesis of exercises previously shown to be effective in managing FHP in the literature [27, 29, 32-34]. "

The authors have no ownership of the intervention platform, and no conflicts of interest are present.

5-ii) Describe the history/development process

Describe the history/development process of the application and previous formative evaluations (e.g., focus groups, usability testing), as these will have an impact on adoption/use rates and help with interpreting results.

	1	2	3	4	5	
subitem not at all important	○	○	○	○	◉	essential

ล้างสิ่งที่เลือก

Does your paper address subitem 5-ii?

Copy and paste relevant sections from the manuscript (include quotes in quotation marks "like this" to indicate direct quotes from your manuscript), or elaborate on this item by providing additional information not in the ms, or briefly explain why the item is not applicable/relevant for your study

"The exercise program was designed by a licensed physiotherapist with 18 years of clinical experience of musculoskeletal rehabilitation, based on a synthesis of exercises previously shown to be effective in managing FHP in the literature [27, 29, 32-34]. "

5-iii) Revisions and updating

Revisions and updating. Clearly mention the date and/or version number of the application/intervention (and comparator, if applicable) evaluated, or describe whether the intervention underwent major changes during the evaluation process, or whether the development and/or content was “frozen” during the trial. Describe dynamic components such as news feeds or changing content which may have an impact on the replicability of the intervention (for unexpected events see item 3b).

	1	2	3	4	5	
subitem not at all important	○	○	○	○	◉	essential

ล้างสิ่งที่เลือก

Does your paper address subitem 5-iii?

Copy and paste relevant sections from the manuscript (include quotes in quotation marks "like this" to indicate direct quotes from your manuscript), or elaborate on this item by providing additional information not in the ms, or briefly explain why the item is not applicable/relevant for your study

he intervention was delivered using the Zoom application, which remained unchanged throughout the trial period. There were no updates, major changes, or dynamic content that could have impacted the intervention delivery.
"The telerehabilitation group performed the exercise program individually under real-time remote supervision in their home environment via secure, password-protected Zoom links. No sessions were recorded, and the Zoom platform remained stable and unchanged during the study. "

5-iv) Quality assurance methods

Provide information on quality assurance methods to ensure accuracy and quality of information provided [1], if applicable.

	1	2	3	4	5	
subitem not at all important	○	○	○	○	◉	essential

ล้างสิ่งที่เลือก

Does your paper address subitem 5-iv?

Copy and paste relevant sections from the manuscript (include quotes in quotation marks "like this" to indicate direct quotes from your manuscript), or elaborate on this item by providing additional information not in the ms, or briefly explain why the item is not applicable/relevant for your study

"The researcher monitored movement patterns and provided verbal feedback to correct exercises, ensuring both proper technique and compliance with the prescribed exercise schedule."

"Therapists followed a standardized session checklist to ensure consistent feedback and technique correction."

5-v) Ensure replicability by publishing the source code, and/or providing screenshots/screen-capture video, and/or providing flowcharts of the algorithms used

Ensure replicability by publishing the source code, and/or providing screenshots/screen-capture video, and/or providing flowcharts of the algorithms used. Replicability (i.e., other researchers should in principle be able to replicate the study) is a hallmark of scientific reporting.

	1	2	3	4	5	
subitem not at all important	○	○	○	○	◉	essential

ล้างสิ่งที่เลือก

Does your paper address subitem 5-v?

Copy and paste relevant sections from the manuscript (include quotes in quotation marks "like this" to indicate direct quotes from your manuscript), or elaborate on this item by providing additional information not in the ms, or briefly explain why the item is not applicable/relevant for your study

We explain the detail of exercises together with pictures in the table. Other researchers should in principle be able to replicate the study.

5-vi) Digital preservation

Digital preservation: Provide the URL of the application, but as the intervention is likely to change or disappear over the course of the years; also make sure the intervention is archived (Internet Archive, webcitation.org, and/or publishing the source code or screenshots/videos alongside the article). As pages behind login screens cannot be archived, consider creating demo pages which are accessible without login.

	1	2	3	4	5	
subitem not at all important	○	○	○	○	◉	essential

ล้างสิ่งที่เลือก

Does your paper address subitem 5-vi?

Copy and paste relevant sections from the manuscript (include quotes in quotation marks "like this" to indicate direct quotes from your manuscript), or elaborate on this item by providing additional information not in the ms, or briefly explain why the item is not applicable/relevant for your study

The intervention was delivered via Zoom, a third-party videoconferencing platform. As Zoom is not custom-developed for this study, no specific application URL is applicable.

5-vii) Access

Access: Describe how participants accessed the application, in what setting/context, if they had to pay (or were paid) or not, whether they had to be a member of specific group. If known, describe how participants obtained “access to the platform and Internet” [1]. To ensure access for editors/reviewers/readers, consider to provide a “backdoor” login account or demo mode for reviewers/readers to explore the application (also important for archiving purposes, see vi).

	1	2	3	4	5	
subitem not at all important	○	○	○	○	◉	essential

ล้างสิ่งที่เลือก

Does your paper address subitem 5-vii? *

Copy and paste relevant sections from the manuscript (include quotes in quotation marks "like this" to indicate direct quotes from your manuscript), or elaborate on this item by providing additional information not in the ms, or briefly explain why the item is not applicable/relevant for your study

Participants accessed the intervention through the Zoom application using their personal devices at home. Access was provided via scheduled meeting links sent by the research team. There were no costs incurred by the participants, nor were they paid to participate. No specific group membership was required to join. All participants had to have access to a smartphone or computer with internet connectivity, typically through their home Wi-Fi or mobile data. Since Zoom is a third-party platform and not custom-developed, no demo account or backdoor access is applicable.

5-viii) Mode of delivery, features/functionalities/components of the intervention and comparator, and the theoretical framework

Describe mode of delivery, features/functionalities/components of the intervention and comparator, and the theoretical framework [6] used to design them (instructional strategy [1], behaviour change techniques, persuasive features, etc., see e.g., [7, 8] for terminology). This includes an in-depth description of the content (including where it is coming from and who developed it) [1],” whether [and how] it is tailored to individual circumstances and allows users to track their progress and receive feedback” [6]. This also includes a description of communication delivery channels and – if computer-mediated communication is a component – whether communication was synchronous or asynchronous [6]. It also includes information on presentation strategies [1], including page design principles, average amount of text on pages, presence of hyperlinks to other resources, etc. [1].

	1	2	3	4	5	
subitem not at all important	○	○	○	○	◉	essential

ล้างสิ่งที่เลือก

Does your paper address subitem 5-viii? *

Copy and paste relevant sections from the manuscript (include quotes in quotation marks "like this" to indicate direct quotes from your manuscript), or elaborate on this item by providing additional information not in the ms, or briefly explain why the item is not applicable/relevant for your study

"Following the initial randomization meeting, all participants received patient education regarding postural correction and guidance on avoiding certain activities, particularly rest break guidelines for mobile electronic device and computer use [32]. Both groups received the same exercise program; a combined stretching and strengthening corrective exercise intervention conducted three times per week for six weeks. The exercise program was designed by a licensed physiotherapist with 18 years of clinical experience of musculoskeletal rehabilitation, based on a synthesis of exercises previously shown to be effective in managing FHP in the literature [27, 29, 32-34]. Participants were instructed to stretch each muscle, including the pectoralis, sternocleidomastoid, upper trapezius, and levator scapulae, to the end of its range without inducing pain or discomfort, following standard protocols. Each stretch was held for 30 seconds and repeated twice for each muscle on both the right and left sides, with a 30-second rest period between repetitions and between muscles. Four strengthening corrective exercises were performed: sitting chin tuck, standing W-to-Y, wall slide with shoulder flexion while facing the wall, and wall slide with shoulder abduction and external rotation while positioned with the back against the wall. Each exercise consisted of 10 repetitions per set, with three sets completed for each exercise. A one-minute rest period was provided between sets, and a two-second rest was allowed between exercises. Each session lasted approximately 40 minutes. The exercise protocol and remote supervision model remained unchanged throughout the trial. Participants in both groups received weekly reminders via the LINE application before each scheduled session. The detailed exercise prescription for the strengthening corrective exercises is provided in Multimedia Appendix 1.
The telerehabilitation group performed the exercise program individually under real-time remote supervision in their home environment via secure, password-protected Zoom links. No sessions were recorded, and the Zoom platform remained stable and unchanged during the study. The researcher monitored movement patterns and provided verbal feedback to correct exercises, ensuring both proper technique and compliance with the prescribed exercise schedule.
The in-person group completed the same program under supervision at a physiotherapy laboratory. Another researcher monitored the movement patterns and provided both verbal instructions and manual correction when necessary. This difference in feedback modality was intentional and designed to reflect real-world remote rehabilitation settings, where tactile feedback is not feasible. Therapists followed a standardized session checklist to ensure consistent feedback and technique correction."

5-ix) Describe use parameters

Describe use parameters (e.g., intended “doses” and optimal timing for use). Clarify what instructions or recommendations were given to the user, e.g., regarding timing, frequency, heaviness of use, if any, or was the intervention used ad libitum.

	1	2	3	4	5	
subitem not at all important	○	○	○	○	◉	essential

ล้างสิ่งที่เลือก

Does your paper address subitem 5-ix?

Copy and paste relevant sections from the manuscript (include quotes in quotation marks "like this" to indicate direct quotes from your manuscript), or elaborate on this item by providing additional information not in the ms, or briefly explain why the item is not applicable/relevant for your study

"Both groups received the same exercise program; a combined stretching and strengthening corrective exercise intervention conducted three times per week for six weeks."
"Each stretch was held for 30 seconds and repeated twice for each muscle on both the right and left sides, with a 30-second rest period between repetitions and between muscles."
"Each exercise consisted of 10 repetitions per set, with three sets completed for each exercise. A one-minute rest period was provided between sets, and a two-second rest was allowed between exercises."
"Each session lasted approximately 40 minutes."

5-x) Clarify the level of human involvement

Clarify the level of human involvement (care providers or health professionals, also technical assistance) in the e-intervention or as co-intervention (detail number and expertise of professionals involved, if any, as well as “type of assistance offered, the timing and frequency of the support, how it is initiated, and the medium by which the assistance is delivered”. It may be necessary to distinguish between the level of human involvement required for the trial, and the level of human involvement required for a routine application outside of a RCT setting (discuss under item 21 – generalizability).

	1	2	3	4	5	
subitem not at all important	○	○	○	○	◉	essential

ล้างสิ่งที่เลือก

Does your paper address subitem 5-x?

Copy and paste relevant sections from the manuscript (include quotes in quotation marks "like this" to indicate direct quotes from your manuscript), or elaborate on this item by providing additional information not in the ms, or briefly explain why the item is not applicable/relevant for your study

"The telerehabilitation group performed the exercise program individually under real-time remote supervision in their home environment via secure, password-protected Zoom links. No sessions were recorded, and the Zoom platform remained stable and unchanged during the study. The researcher monitored movement patterns and provided verbal feedback to correct exercises, ensuring both proper technique and compliance with the prescribed exercise schedule."
"The in-person group completed the same program under supervision at a physiotherapy laboratory. Another researcher monitored the movement patterns and provided both verbal instructions and manual correction when necessary. "

5-xi) Report any prompts/reminders used

Report any prompts/reminders used: Clarify if there were prompts (letters, emails, phone calls, SMS) to use the application, what triggered them, frequency etc. It may be necessary to distinguish between the level of prompts/reminders required for the trial, and the level of prompts/reminders for a routine application outside of a RCT setting (discuss under item 21 – generalizability).

	1	2	3	4	5	
subitem not at all important	○	○	○	○	◉	essential

ล้างสิ่งที่เลือก

Does your paper address subitem 5-xi? *

Copy and paste relevant sections from the manuscript (include quotes in quotation marks "like this" to indicate direct quotes from your manuscript), or elaborate on this item by providing additional information not in the ms, or briefly explain why the item is not applicable/relevant for your study

"Participants in both groups received weekly reminders via the LINE application before each scheduled session. "

5-xii) Describe any co-interventions (incl. training/support)

Describe any co-interventions (incl. training/support): Clearly state any interventions that are provided in addition to the targeted eHealth intervention, as ehealth intervention may not be designed as stand-alone intervention. This includes training sessions and support [1]. It may be necessary to distinguish between the level of training required for the trial, and the level of training for a routine application outside of a RCT setting (discuss under item 21 – generalizability.

	1	2	3	4	5	
subitem not at all important	○	○	◉	○	○	essential

ล้างสิ่งที่เลือก

Does your paper address subitem 5-xii? *

Copy and paste relevant sections from the manuscript (include quotes in quotation marks "like this" to indicate direct quotes from your manuscript), or elaborate on this item by providing additional information not in the ms, or briefly explain why the item is not applicable/relevant for your study

"all participants received patient education regarding postural correction and guidance on avoiding certain activities, particularly rest break guidelines for mobile electronic device and computer use [32]. "

6a) Completely defined pre-specified primary and secondary outcome measures, including how and when they were assessed

Does your paper address CONSORT subitem 6a? *

Copy and paste relevant sections from the manuscript (include quotes in quotation marks "like this" to indicate direct quotes from your manuscript), or elaborate on this item by providing additional information not in the ms, or briefly explain why the item is not applicable/relevant for your study

"Neck pain, CVA, neck disability level, and neck range of motion (ROM) were evaluated at baseline, after four weeks of intervention, after six weeks of intervention, and during a two-week follow-up period. Evaluations were administered in-person in the physiotherapy department laboratory by an assessor who was blinded to group allocation and not involved in the intervention for either group. No modifications were made to the trial outcomes after the commencement of the trial. The outcomes outlined in the protocol remained consistent throughout the study period.
Neck pain was assessed using the VAS within a 24-hour period. The VAS, widely regarded as one of the most reliable and validated methods for self-reporting pain intensity, utilizes a 10 cm horizontal line. On this scale, 0 cm indicates "No pain", while 10 cm represents "Worst pain" [28].
CVA was measured using photogrammetry in the sitting position. Markers were placed over the right tragus of the ear and the C7 spinous process. Participants were instructed to sit in their neutral resting position with their arms naturally on their knees [29]. A digital camera was positioned on a tripod at shoulder level, 0.8 meters away from the participants. Angles were measured using Kinovea, a 2D motion analysis software. CVA was measured from the intersection between a horizontal line through the spinous process of C7 and a line extending to the tragus of the ear. Each angle was measured three times to enhance data reliability.
Neck disability level was determined using the Neck Disability Index (NDI), Thai Version. This self-reported questionnaire determined the disability index of the cervical spine and its impact on daily activities. The NDI has been validated as an effective evaluative tool for patients with neck pain [30].
Neck ROM was measured using a CROM device (Performance Attainment Associates and MedNet Technologies, Inc. USA). Active ROM of the neck was assessed in six directions: flexion, extension, lateral flexion to the left, lateral flexion to the right, rotation to the left, and rotation to the right. The device has demonstrated high intra-tester reliability (intra-class correlation coefficient (ICC) = 0.87–0.96) for patients with neck pain [31]."

6a-i) Online questionnaires: describe if they were validated for online use and apply CHERRIES items to describe how the questionnaires were designed/deployed

If outcomes were obtained through online questionnaires, describe if they were validated for online use and apply CHERRIES items to describe how the questionnaires were designed/deployed [9].

	1	2	3	4	5	
subitem not at all important	○	○	◉	○	○	essential

ล้างสิ่งที่เลือก

Does your paper address subitem 6a-i?

Copy and paste relevant sections from manuscript text

Not applicable. No online questionnaire was used.

6a-ii) Describe whether and how “use” (including intensity of use/dosage) was defined/measured/monitored

Describe whether and how “use” (including intensity of use/dosage) was defined/measured/monitored (logins, logfile analysis, etc.). Use/adoption metrics are important process outcomes that should be reported in any ehealth trial.

	1	2	3	4	5	
subitem not at all important	○	○	○	○	◉	essential

ล้างสิ่งที่เลือก

Does your paper address subitem 6a-ii?

Copy and paste relevant sections from manuscript text

"exercise intervention conducted three times per week for six weeks. "
"Each session lasted approximately 40 minutes."
"Adherence to the intervention was monitored in both groups using attendance logs and considered as a process outcome. All scheduled sessions were monitored, and participant adherence was defined by session attendance. "

6a-iii) Describe whether, how, and when qualitative feedback from participants was obtained

Describe whether, how, and when qualitative feedback from participants was obtained (e.g., through emails, feedback forms, interviews, focus groups).

	1	2	3	4	5	
subitem not at all important	○	○	○	●	○	essential

ล้างสิ่งที่เลือก

Does your paper address subitem 6a-iii?

Copy and paste relevant sections from manuscript text

No qualitative feedback regarding participant satisfaction or experience was collected during the study.

6b) Any changes to trial outcomes after the trial commenced, with reasons

Does your paper address CONSORT subitem 6b? *

Copy and paste relevant sections from the manuscript (include quotes in quotation marks "like this" to indicate direct quotes from your manuscript), or elaborate on this item by providing additional information not in the ms, or briefly explain why the item is not applicable/relevant for your study

"No modifications were made to the trial outcomes after the commencement of the trial. The outcomes outlined in the protocol remained consistent throughout the study period."

7a) How sample size was determined

NPT: When applicable, details of whether and how the clustering by care provides or centers was addressed

7a-i) Describe whether and how expected attrition was taken into account when calculating the sample size

Describe whether and how expected attrition was taken into account when calculating the sample size.

	1	2	3	4	5	
subitem not at all important	○	○	○	○	●	essential

ล้างสิ่งที่เลือก

Does your paper address subitem 7a-i?

Copy and paste relevant sections from manuscript title (include quotes in quotation marks "like this" to indicate direct quotes from your manuscript), or elaborate on this item by providing additional information not in the ms, or briefly explain why the item is not applicable/relevant for your study

An a priori power analysis was conducted using G*Power software (version 3.1.9.7, Germany) to determine the required sample size. The calculation was based on an F-test using the effect size reported in a previous study comparing therapeutic exercises with a control group [27]. A total of 42 participants was determined through the calculations. However, a 15 percent dropout rate was anticipated. As a result, the final target sample size for this study was 50 participants.

7b) When applicable, explanation of any interim analyses and stopping guidelines

Does your paper address CONSORT subitem 7b? *

Copy and paste relevant sections from the manuscript (include quotes in quotation marks "like this" to indicate direct quotes from your manuscript), or elaborate on this item by providing additional information not in the ms, or briefly explain why the item is not applicable/relevant for your study

"No interim analyses were performed during the trial, and stopping guidelines were not relevant. The trial adhered strictly to the predetermined protocol without any deviations concerning interim analyses or early termination."

8a) Method used to generate the random allocation sequence

NPT: When applicable, how care providers were allocated to each trial group

Does your paper address CONSORT subitem 8a? *

Copy and paste relevant sections from the manuscript (include quotes in quotation marks "like this" to indicate direct quotes from your manuscript), or elaborate on this item by providing additional information not in the ms, or briefly explain why the item is not applicable/relevant for your study

"Participants were randomly assigned to either the telerehabilitation or in-person group through a concealed allocation process. At the beginning of the program, an independent researcher, not involved in any assessments, prepared 50 small opaque slips labeled “1” and “2”. Each participant drew one slip from a container to determine their group assignment. To ensure allocation concealment, sealed and sequentially numbered opaque containers were used, thereby preserving the integrity of the randomization process and minimizing the risk of selection bias. "

8b) Type of randomisation; details of any restriction (such as blocking and block size)

Does your paper address CONSORT subitem 8b? *

Copy and paste relevant sections from the manuscript (include quotes in quotation marks "like this" to indicate direct quotes from your manuscript), or elaborate on this item by providing additional information not in the ms, or briefly explain why the item is not applicable/relevant for your study

"Participants were randomly assigned to either the telerehabilitation or in-person group through a concealed allocation process. At the beginning of the program, an independent researcher, not involved in any assessments, prepared 50 small opaque slips labeled “1” and “2”. Each participant drew one slip from a container to determine their group assignment. To ensure allocation concealment, sealed and sequentially numbered opaque containers were used, thereby preserving the integrity of the randomization process and minimizing the risk of selection bias."

9) Mechanism used to implement the random allocation sequence (such as sequentially numbered containers), describing any steps taken to conceal the sequence until interventions were assigned

Does your paper address CONSORT subitem 9? *

Copy and paste relevant sections from the manuscript (include quotes in quotation marks "like this" to indicate direct quotes from your manuscript), or elaborate on this item by providing additional information not in the ms, or briefly explain why the item is not applicable/relevant for your study

" prepared 50 small opaque slips labeled “1” and “2”. Each participant drew one slip from a container to determine their group assignment. To ensure allocation concealment, sealed and sequentially numbered opaque containers were used, thereby preserving the integrity of the randomization process and minimizing the risk of selection bias. "

10) Who generated the random allocation sequence, who enrolled participants, and who assigned participants to interventions

Does your paper address CONSORT subitem 10? *

Copy and paste relevant sections from the manuscript (include quotes in quotation marks "like this" to indicate direct quotes from your manuscript), or elaborate on this item by providing additional information not in the ms, or briefly explain why the item is not applicable/relevant for your study

"At the beginning of the program, an independent researcher, not involved in any assessments, prepared 50 small opaque slips labeled “1” and “2”. "

11a) If done, who was blinded after assignment to interventions (for example, participants, care providers, those assessing outcomes) and how

NPT: Whether or not administering co-interventions were blinded to group assignment

11a-i) Specify who was blinded, and who wasn’t

Specify who was blinded, and who wasn’t. Usually, in web-based trials it is not possible to blind the participants [1, 3] (this should be clearly acknowledged), but it may be possible to blind outcome assessors, those doing data analysis or those administering co-interventions (if any).

	1	2	3	4	5	
subitem not at all important	○	○	○	○	◉	essential

ล้างสิ่งที่เลือก

Does your paper address subitem 11a-i? *

Copy and paste relevant sections from the manuscript (include quotes in quotation marks "like this" to indicate direct quotes from your manuscript), or elaborate on this item by providing additional information not in the ms, or briefly explain why the item is not applicable/relevant for your study

"At the beginning of the program, an independent researcher, not involved in any assessments, prepared 50 small opaque slips labeled “1” and “2”."
"The outcome assessors were blinded to group assignments throughout the study. "

11a-ii) Discuss e.g., whether participants knew which intervention was the "intervention of interest" and which one was the "comparator"

Informed consent procedures (4a-ii) can create biases and certain expectations - discuss e.g., whether participants knew which intervention was the "intervention of interest" and which one was the "comparator".

	1	2	3	4	5	
subitem not at all important	○	○	○	○	◉	essential

ล้างสิ่งที่เลือก

Does your paper address subitem 11a-ii?

Copy and paste relevant sections from the manuscript (include quotes in quotation marks "like this" to indicate direct quotes from your manuscript), or elaborate on this item by providing additional information not in the ms, or briefly explain why the item is not applicable/relevant for your study

During the informed consent process, participants were informed that the study aimed to compare two modes of exercise delivery—telerehabilitation and in-person supervision. While the purpose of comparison was explained, participants were not explicitly told which intervention was considered the primary intervention of interest.
"Both groups received the same exercise program; a combined stretching and strengthening corrective exercise intervention."

11b) If relevant, description of the similarity of interventions

(this item is usually not relevant for ehealth trials as it refers to similarity of a placebo or sham intervention to a active medication/intervention)

Does your paper address CONSORT subitem 11b? *

Copy and paste relevant sections from the manuscript (include quotes in quotation marks "like this" to indicate direct quotes from your manuscript), or elaborate on this item by providing additional information not in the ms, or briefly explain why the item is not applicable/relevant for your study

This item is not applicable to the present study. The interventions (telerehabilitation vs. in-person supervision) were clearly distinct in format and delivery method, and no placebo or sham intervention was used

12a) Statistical methods used to compare groups for primary and secondary outcomes

NPT: When applicable, details of whether and how the clustering by care providers or centers was addressed

Does your paper address CONSORT subitem 12a? *

Copy and paste relevant sections from the manuscript (include quotes in quotation marks "like this" to indicate direct quotes from your manuscript), or elaborate on this item by providing additional information not in the ms, or briefly explain why the item is not applicable/relevant for your study

"Statistical analysis was conducted using SPSS for Windows version 23 (IBM Corp., Armonk, NY, USA). A p-value of .05 was considered statistically significant. Descriptive statistics, including the mean, standard deviation (SD), and 95% confidence interval (CI), were reported. Intervention effects on outcome measures were assessed via repeated-measures analysis of variance with a 2-by-4 design (two groups and four time points). The effect size was indicated by partial eta squared (η^2). In the case of significant group-by-time interactions, post-hoc pairwise comparisons with Bonferroni correction were used to adjust for multiple comparisons."

12a-i) Imputation techniques to deal with attrition / missing values

Imputation techniques to deal with attrition / missing values: Not all participants will use the intervention/comparator as intended and attrition is typically high in ehealth trials. Specify how participants who did not use the application or dropped out from the trial were treated in the statistical analysis (a complete case analysis is strongly discouraged, and simple imputation techniques such as LOCF may also be problematic [4]).

	1	2	3	4	5	
subitem not at all important	○	○	○	○	◉	essential

ล้างสิ่งที่เลือก

Does your paper address subitem 12a-i? *

Copy and paste relevant sections from the manuscript (include quotes in quotation marks "like this" to indicate direct quotes from your manuscript), or elaborate on this item by providing additional information not in the ms, or briefly explain why the item is not applicable/relevant for your study

"Intention-to-treat analysis was applied to account for altered results from baseline if data were lost during follow-up."

12b) Methods for additional analyses, such as subgroup analyses and adjusted analyses

Does your paper address CONSORT subitem 12b? *

Copy and paste relevant sections from the manuscript (include quotes in quotation marks "like this" to indicate direct quotes from your manuscript), or elaborate on this item by providing additional information not in the ms, or briefly explain why the item is not applicable/relevant for your study

"No additional analyses, such as subgroup analyses or adjusted analyses, were conducted. The baseline characteristics were similar between the groups for all variables."

X26) REB/IRB Approval and Ethical Considerations [recommended as subheading under "Methods"] (not a CONSORT item)

X26-i) Comment on ethics committee approval

	1	2	3	4	5	
subitem not at all important	○	○	○	○	◉	essential

ล้างสิ่งที่เลือก

Does your paper address subitem X26-i?

Copy and paste relevant sections from the manuscript (include quotes in quotation marks "like this" to indicate direct quotes from your manuscript), or elaborate on this item by providing additional information not in the ms, or briefly explain why the item is not applicable/relevant for your study

"The study protocol was approved by the Human Research Ethics Committee of Walailak University, Thailand (Approval number: WUEC-23-335-02)"

x26-ii) Outline informed consent procedures

Outline informed consent procedures e.g., if consent was obtained offline or online (how? Checkbox, etc.?), and what information was provided (see 4a-ii). See [6] for some items to be included in informed consent documents.

	1	2	3	4	5	
subitem not at all important	○	○	○	○	◉	essential

ล้างสิ่งที่เลือก

Does your paper address subitem X26-ii?

Copy and paste relevant sections from the manuscript (include quotes in quotation marks "like this" to indicate direct quotes from your manuscript), or elaborate on this item by providing additional information not in the ms, or briefly explain why the item is not applicable/relevant for your study

"Written informed consent was obtained from all participants. "

X26-iii) Safety and security procedures

Safety and security procedures, incl. privacy considerations, and any steps taken to reduce the likelihood or detection of harm (e.g., education and training, availability of a hotline)

	1	2	3	4	5	
subitem not at all important	○	○	○	○	◉	essential

ล้างสิ่งที่เลือก

Does your paper address subitem X26-iii?

Copy and paste relevant sections from the manuscript (include quotes in quotation marks "like this" to indicate direct quotes from your manuscript), or elaborate on this item by providing additional information not in the ms, or briefly explain why the item is not applicable/relevant for your study

"The telerehabilitation group performed the exercise program individually under real-time remote supervision in their home environment via secure, password-protected Zoom links. No sessions were recorded, "

RESULTS

13a) For each group, the numbers of participants who were randomly assigned, received intended treatment, and were analysed for the primary outcome

NPT: The number of care providers or centers performing the intervention in each group and the number of patients treated by each care provider in each center

Does your paper address CONSORT subitem 13a? *

Copy and paste relevant sections from the manuscript (include quotes in quotation marks "like this" to indicate direct quotes from your manuscript), or elaborate on this item by providing additional information not in the ms, or briefly explain why the item is not applicable/relevant for your study

"This study initially recruited 72 participants for screening, of which 22 were disqualified, leaving 50 participants enrolled. A total 50 participants were randomized into telerehabilitation and in-person groups. A depiction of a participant in the trial is presented in Figure 1. "

13b) For each group, losses and exclusions after randomisation, together with reasons

Does your paper address CONSORT subitem 13b? (NOTE: Preferably, this is shown in a CONSORT flow diagram) *

Copy and paste relevant sections from the manuscript (include quotes in quotation marks "like this" to indicate direct quotes from your manuscript), or elaborate on this item by providing additional information not in the ms, or briefly explain why the item is not applicable/relevant for your study

"This study initially recruited 72 participants for screening, of which 22 were disqualified, leaving 50 participants enrolled."

13b-i) Attrition diagram

Strongly recommended: An attrition diagram (e.g., proportion of participants still logging in or using the intervention/comparator in each group plotted over time, similar to a survival curve) or other figures or tables demonstrating usage/dose/engagement.

	1	2	3	4	5	
subitem not at all important	○	○	○	○	◉	essential

ล้างสิ่งที่เลือก

Does your paper address subitem 13b-i?

Copy and paste relevant sections from the manuscript or cite the figure number if applicable (include quotes in quotation marks "like this" to indicate direct quotes from your manuscript), or elaborate on this item by providing additional information not in the ms, or briefly explain why the item is not applicable/relevant for your study

"A depiction of a participant in the trial is presented in Figure 1. "

14a) Dates defining the periods of recruitment and follow-up

Does your paper address CONSORT subitem 14a? *

Copy and paste relevant sections from the manuscript (include quotes in quotation marks "like this" to indicate direct quotes from your manuscript), or elaborate on this item by providing additional information not in the ms, or briefly explain why the item is not applicable/relevant for your study

"Mean and standard deviation (SD) of Visual Analog Scale (VAS), Craniovertebral angle (CVA), neck disability index (NDI), and Cervical range of motion (CROM) at baseline, after four weeks of intervention, after six weeks of intervention, and during a two-week follow-up period of both telerehabilitation and in-person groups were presented in Table 2."

14a-i) Indicate if critical “secular events” fell into the study period

Indicate if critical “secular events” fell into the study period, e.g., significant changes in Internet resources available or “changes in computer hardware or Internet delivery resources”

	1	2	3	4	5	
subitem not at all important	○	○	○	○	●	essential

ล้างสิ่งที่เลือก

Does your paper address subitem 14a-i?

Copy and paste relevant sections from the manuscript (include quotes in quotation marks "like this" to indicate direct quotes from your manuscript), or elaborate on this item by providing additional information not in the ms, or briefly explain why the item is not applicable/relevant for your study

"No harm or unintended effects were observed in either group. The participants reported no adverse events during the treatment period, nor were any identified during the two-week follow-up."

14b) Why the trial ended or was stopped (early)

Does your paper address CONSORT subitem 14b? *

Copy and paste relevant sections from the manuscript (include quotes in quotation marks "like this" to indicate direct quotes from your manuscript), or elaborate on this item by providing additional information not in the ms, or briefly explain why the item is not applicable/relevant for your study

The trial was completed as planned and was not stopped early.

15) A table showing baseline demographic and clinical characteristics for each group

NPT: When applicable, a description of care providers (case volume, qualification, expertise, etc.) and centers (volume) in each group

Does your paper address CONSORT subitem 15? *

Copy and paste relevant sections from the manuscript (include quotes in quotation marks "like this" to indicate direct quotes from your manuscript), or elaborate on this item by providing additional information not in the ms, or briefly explain why the item is not applicable/relevant for your study

"The participants' characteristics are presented in Table 1."
"Table 2. Mean (SD) score by group over time."
"Table 3. Mean (95% CI) change within group over time."

15-i) Report demographics associated with digital divide issues

In ehealth trials it is particularly important to report demographics associated with digital divide issues, such as age, education, gender, social-economic status, computer/Internet/ehealth literacy of the participants, if known.

	1	2	3	4	5	
subitem not at all important	○	○	○	○	◉	essential

ล้างสิ่งที่เลือก

Does your paper address subitem 15-i? *

Copy and paste relevant sections from the manuscript (include quotes in quotation marks "like this" to indicate direct quotes from your manuscript), or elaborate on this item by providing additional information not in the ms, or briefly explain why the item is not applicable/relevant for your study

"The participants' characteristics are presented in Table 1."

16) For each group, number of participants (denominator) included in each analysis and whether the analysis was by original assigned groups

16-i) Report multiple "denominators" and provide definitions

Report multiple "denominators" and provide definitions: Report N's (and effect sizes) "across a range of study participation [and use] thresholds" [1], e.g., N exposed, N consented, N used more than x times, N used more than y weeks, N participants "used" the intervention/comparator at specific pre-defined time points of interest (in absolute and relative numbers per group). Always clearly define "use" of the intervention.

	1	2	3	4	5	
subitem not at all important	○	○	○	○	◉	essential

ล้างสิ่งที่เลือก

Does your paper address subitem 16-i? *

Copy and paste relevant sections from the manuscript (include quotes in quotation marks "like this" to indicate direct quotes from your manuscript), or elaborate on this item by providing additional information not in the ms, or briefly explain why the item is not applicable/relevant for your study

"This study initially recruited 72 participants for screening, of which 22 were disqualified, leaving 50 participants enrolled. A total 50 participants were randomized into telerehabilitation and in-person groups. A depiction of a participant in the trial is presented in Figure 1."
"One participant from telerehabilitation group and another participant from in-person group withdrew, leading to drop out. Among the 48 participants who completed the intervention, adherence was 100% (48/48), with all participants attending every scheduled session over the six-week period. "
"The intention-to-treat principle was used, and all 50 participants were included in the analysis (Figure 1)."

16-ii) Primary analysis should be intent-to-treat

Primary analysis should be intent-to-treat, secondary analyses could include comparing only “users”, with the appropriate caveats that this is no longer a randomized sample (see 18-i).

	1	2	3	4	5	
subitem not at all important	○	○	○	○	◉	essential

ล้างสิ่งที่เลือก

Does your paper address subitem 16-ii?

Copy and paste relevant sections from the manuscript (include quotes in quotation marks "like this" to indicate direct quotes from your manuscript), or elaborate on this item by providing additional information not in the ms, or briefly explain why the item is not applicable/relevant for your study

"The intention-to-treat principle was used, and all 50 participants were included in the analysis (Figure 1)."

17a) For each primary and secondary outcome, results for each group, and the estimated effect size and its precision (such as 95% confidence interval)

Does your paper address CONSORT subitem 17a? *

Copy and paste relevant sections from the manuscript (include quotes in quotation marks "like this" to indicate direct quotes from your manuscript), or elaborate on this item by providing additional information not in the ms, or briefly explain why the item is not applicable/relevant for your study

"Mean and standard deviation (SD) of Visual Analog Scale (VAS), Craniovertebral angle (CVA), neck disability index (NDI), and Cervical range of motion (CROM) at baseline, after four weeks of intervention, after six weeks of intervention, and during a two-week follow-up period of both telerehabilitation and in-person groups were presented in Table 2."

"The multivariate test demonstrated that the main effect of group-by-time interaction had no significant difference of all variables including VAS ($F_{3,46}$=1.001, P=.40, η^2=0.061), CVA ($F_{3,46}$=1.554, P=.21, η^2=0.092), NDI ($F_{3,46}$=1.489, P=.23, η^2=0.089), CROM of flexion ($F_{3,46}$=0.610, P=.61, η^2=0.038), CROM of extension ($F_{3,46}$=2.181, P=.10, η^2=0.125), CROM of lateral flexion to left ($F_{3,46}$=1.633, P=.19, η^2=0.096), CROM of lateral flexion to right ($F_{3,46}$=2.529, P=.07, η^2=0.142), CROM of rotation to left ($F_{3,46}$=1.102, P=.36, η^2=0.067), and CROM of rotation to right ($F_{3,46}$=1.048, P=.38, η^2=0.064)."
"However, the main effect of time was statistical significant in all parameters; VAS ($F_{3,46}$=85.673, P<.001, η^2=0.848), CVA ($F_{3,46}$=69.383, P<.001, η^2=0.819), NDI ($F_{3,46}$=49.704, P<.001, η^2=0.764), CROM of flexion ($F_{3,46}$=61.035, P<.001, η^2=0.799), CROM of extension ($F_{3,46}$=35.699, P<.001, η^2=0.700), CROM of lateral flexion to left ($F_{3,46}$=86.050, P<.001, η^2=0.849), CROM of lateral flexion to right ($F_{3,46}$=62.989, P<.001, η^2=0.804), CROM of rotation to left ($F_{3,46}$=57.219, P<.001, η^2=0.789), and CROM of rotation to right ($F_{3,46}$=140.378, P<.001, η^2=0.902). "
"Pairwise comparisons using Bonferroni correction revealed statistically significant improvements from baseline (week 0) at all subsequent time points (weeks 4, 6, and 2-week follow-up) in both the telerehabilitation and in-person groups. Significant improvements from baseline were observed in all outcomes, including VAS (P<.001), CVA (P<.001), NDI (P<.001), and CROM in flexion, extension, lateral flexion, and rotation (P<.001 to P=.04, depending on direction and group) (Table 3, Figure 2 and 3)."
"Table 3. Mean (95% CI) change within group over time."

17a-i) Presentation of process outcomes such as metrics of use and intensity of use

In addition to primary/secondary (clinical) outcomes, the presentation of process outcomes such as metrics of use and intensity of use (dose, exposure) and their operational definitions is critical. This does not only refer to metrics of attrition (13-b) (often a binary variable), but also to more continuous exposure metrics such as “average session length”. These must be accompanied by a technical description how a metric like a “session” is defined (e.g., timeout after idle time) [1] (report under item 6a).

	1	2	3	4	5	
subitem not at all important	○	○	○	○	◉	essential

ล้างสิ่งที่เลือก

Does your paper address subitem 17a-i?

Copy and paste relevant sections from the manuscript (include quotes in quotation marks "like this" to indicate direct quotes from your manuscript), or elaborate on this item by providing additional information not in the ms, or briefly explain why the item is not applicable/relevant for your study

"One participant from telerehabilitation group and another participant from in-person group withdrew, leading to drop out. Among the 48 participants who completed the intervention, adherence was 100% (48/48), with all participants attending every scheduled session over the six-week period."

17b) For binary outcomes, presentation of both absolute and relative effect sizes is recommended

Does your paper address CONSORT subitem 17b? *

Copy and paste relevant sections from the manuscript (include quotes in quotation marks "like this" to indicate direct quotes from your manuscript), or elaborate on this item by providing additional information not in the ms, or briefly explain why the item is not applicable/relevant for your study

This item is not applicable, as the study did not include any binary outcome measures.

18) Results of any other analyses performed, including subgroup analyses and adjusted analyses, distinguishing pre-specified from exploratory

Does your paper address CONSORT subitem 18? *

Copy and paste relevant sections from the manuscript (include quotes in quotation marks "like this" to indicate direct quotes from your manuscript), or elaborate on this item by providing additional information not in the ms, or briefly explain why the item is not applicable/relevant for your study

No subgroup, adjusted, or exploratory analyses were performed beyond the primary comparisons specified in the study protocol.

18-i) Subgroup analysis of comparing only users

A subgroup analysis of comparing only users is not uncommon in ehealth trials, but if done, it must be stressed that this is a self-selected sample and no longer an unbiased sample from a randomized trial (see 16-iii).

	1	2	3	4	5	
subitem not at all important	○	○	○	○	◉	essential

ล้างสิ่งที่เลือก

Does your paper address subitem 18-i?

Copy and paste relevant sections from the manuscript (include quotes in quotation marks "like this" to indicate direct quotes from your manuscript), or elaborate on this item by providing additional information not in the ms, or briefly explain why the item is not applicable/relevant for your study

not applicable/relevant for our study. No subgroup analyses.

19) All important harms or unintended effects in each group
(for specific guidance see CONSORT for harms)

Does your paper address CONSORT subitem 19? *

Copy and paste relevant sections from the manuscript (include quotes in quotation marks "like this" to indicate direct quotes from your manuscript), or elaborate on this item by providing additional information not in the ms, or briefly explain why the item is not applicable/relevant for your study

"No harm or unintended effects were observed in either group. The participants reported no adverse events during the treatment period, nor were any identified during the two-week follow-up."

19-i) Include privacy breaches, technical problems

Include privacy breaches, technical problems. This does not only include physical “harm” to participants, but also incidents such as perceived or real privacy breaches [1], technical problems, and other unexpected/unintended incidents. “Unintended effects” also includes unintended positive effects [2].

	1	2	3	4	5	
subitem not at all important	○	○	◉	○	○	essential

ล้างสิ่งที่เลือก

Does your paper address subitem 19-i?

Copy and paste relevant sections from the manuscript (include quotes in quotation marks "like this" to indicate direct quotes from your manuscript), or elaborate on this item by providing additional information not in the ms, or briefly explain why the item is not applicable/relevant for your study

No privacy breaches, technical problems, or unintended effects (positive or negative) were reported during the intervention or follow-up period.

19-ii) Include qualitative feedback from participants or observations from staff/researchers

Include qualitative feedback from participants or observations from staff/researchers, if available, on strengths and shortcomings of the application, especially if they point to unintended/unexpected effects or uses. This includes (if available) reasons for why people did or did not use the application as intended by the developers.

	1	2	3	4	5	
subitem not at all important	○	○	○	○	◉	essential

ล้างสิ่งที่เลือก

Does your paper address subitem 19-ii?

Copy and paste relevant sections from the manuscript (include quotes in quotation marks "like this" to indicate direct quotes from your manuscript), or elaborate on this item by providing additional information not in the ms, or briefly explain why the item is not applicable/relevant for your study

Although no formal qualitative interviews were conducted, informal feedback was collected during and after telerehabilitation sessions. Participants generally reported positive experiences, highlighting the convenience of accessing sessions from home. No major unexpected effects or misuses of the platform were noted.

DISCUSSION

22) Interpretation consistent with results, balancing benefits and harms, and considering other relevant evidence

NPT: In addition, take into account the choice of the comparator, lack of or partial blinding, and unequal expertise of care providers or centers in each group

22-i) Restate study questions and summarize the answers suggested by the data, starting with primary outcomes and process outcomes (use)

Restate study questions and summarize the answers suggested by the data, starting with primary outcomes and process outcomes (use).

	1	2	3	4	5	
subitem not at all important	○	○	○	○	◉	essential

ล้างสิ่งที่เลือก

Does your paper address subitem 22-i? *

Copy and paste relevant sections from the manuscript (include quotes in quotation marks "like this" to indicate direct quotes from your manuscript), or elaborate on this item by providing additional information not in the ms, or briefly explain why the item is not applicable/relevant for your study

"The major finding of this study is that there were no significant differences in neck pain intensity (Visual Analog Scale (VAS)), Craniovertebral angle (CVA), neck disability index (NDI), and Cervical range of motion (CROM) between the telerehabilitation and in-person groups, indicating comparable effectiveness of both delivery methods. Although the group-by-time interaction was not statistically significant, the partial eta squared values supported the overall interpretation that both telerehabilitation and in-person exercise produced clinically meaningful improvements. The consistently large effect sizes observed for the main effect of time further emphasize the effectiveness of the intervention across both delivery methods. In addition, adherence to the intervention was excellent, with all participants who completed the program attending all scheduled sessions. This high adherence rate demonstrates the feasibility and acceptance of both approaches among participants. These findings support the clinical viability of remote rehabilitation models when implemented with appropriate supervision and technological support."

22-ii) Highlight unanswered new questions, suggest future research

Highlight unanswered new questions, suggest future research.

	1	2	3	4	5	
subitem not at all important	○	○	○	○	◉	essential

ล้างสิ่งที่เลือก

Does your paper address subitem 22-ii?

Copy and paste relevant sections from the manuscript (include quotes in quotation marks "like this" to indicate direct quotes from your manuscript), or elaborate on this item by providing additional information not in the ms, or briefly explain why the item is not applicable/relevant for your study

"Future research should explore the long-term sustainability of treatment effects, cost effectiveness, and broader applicability of telerehabilitation across diverse populations and clinical settings. To better understand treatment response and optimize intervention strategies, future studies should incorporate subgroup analyses based on baseline characteristics such as pain duration and include objective measures of cervical and upper body muscle strength to more accurately assess the physiological impact of corrective exercises."

20) Trial limitations, addressing sources of potential bias, imprecision, and, if relevant, multiplicity of analyses

20-i) Typical limitations in ehealth trials

Typical limitations in ehealth trials: Participants in ehealth trials are rarely blinded. Ehealth trials often look at a multiplicity of outcomes, increasing risk for a Type I error. Discuss biases due to non-use of the intervention/usability issues, biases through informed consent procedures, unexpected events.

	1	2	3	4	5	
subitem not at all important	○	○	○	○	◉	essential

ล้างสิ่งที่เลือก

Does your paper address subitem 20-i? *

Copy and paste relevant sections from the manuscript (include quotes in quotation marks "like this" to indicate direct quotes from your manuscript), or elaborate on this item by providing additional information not in the ms, or briefly explain why the item is not applicable/relevant for your study

"This study had some limitations. First, exercises performed in a lying position were excluded because telerehabilitation required participants to remain in front of a mobile device or laptop for the physiotherapist to observe and provide feedback effectively. "
"Third, although participant blinding was not feasible due to the nature of the intervention, this is a common limitation in exercise trials. "

21) Generalisability (external validity, applicability) of the trial findings

NPT: External validity of the trial findings according to the intervention, comparators, patients, and care providers or centers involved in the trial

21-i) Generalizability to other populations

Generalizability to other populations: In particular, discuss generalizability to a general Internet population, outside of a RCT setting, and general patient population, including applicability of the study results for other organizations

	1	2	3	4	5	
subitem not at all important	○	○	○	○	◉	essential

ล้างสิ่งที่เลือก

Does your paper address subitem 21-i?

Copy and paste relevant sections from the manuscript (include quotes in quotation marks "like this" to indicate direct quotes from your manuscript), or elaborate on this item by providing additional information not in the ms, or briefly explain why the item is not applicable/relevant for your study

"the study focused on young adult females, the findings may not be generalizable to individuals of other sexes or age ranges. These limitations suggest that caution should be exercised when applying the findings to broader patient populations, particularly to males, older adults, or those requiring long-term care"

21-ii) Discuss if there were elements in the RCT that would be different in a routine application setting

Discuss if there were elements in the RCT that would be different in a routine application setting (e.g., prompts/reminders, more human involvement, training sessions or other co-interventions) and what impact the omission of these elements could have on use, adoption, or outcomes if the intervention is applied outside of a RCT setting.

	1	2	3	4	5	
subitem not at all important	○	○	◉	○	○	essential

ล้างสิ่งที่เลือก

Does your paper address subitem 21-ii?

Copy and paste relevant sections from the manuscript (include quotes in quotation marks "like this" to indicate direct quotes from your manuscript), or elaborate on this item by providing additional information not in the ms, or briefly explain why the item is not applicable/relevant for your study

This item is not directly addressed in the manuscript. However, we acknowledge that certain elements in the RCT—such as therapist-led session scheduling, real-time supervision, and consistent motivational support—may not be present in routine clinical settings. These factors could influence adherence and outcomes when the intervention is implemented in real-world practice. Additional strategies such as automated reminders or app-based support may be useful to bridge this gap in future applications.

OTHER INFORMATION

23) Registration number and name of trial registry

Does your paper address CONSORT subitem 23? *

Copy and paste relevant sections from the manuscript (include quotes in quotation marks "like this" to indicate direct quotes from your manuscript), or elaborate on this item by providing additional information not in the ms, or briefly explain why the item is not applicable/relevant for your study

"the trial was registered with the Thai Clinical Trials Registry (Registration number: TCTR20240326007). "

24) Where the full trial protocol can be accessed, if available

Does your paper address CONSORT subitem 24? *

Cite a Multimedia Appendix, other reference, or copy and paste relevant sections from the manuscript (include quotes in quotation marks "like this" to indicate direct quotes from your manuscript), or elaborate on this item by providing additional information not in the ms, or briefly explain why the item is not applicable/relevant for your study

The full trial protocol is not published online but is available upon request from the corresponding author.

25) Sources of funding and other support (such as supply of drugs), role of funders

Does your paper address CONSORT subitem 25? *

Copy and paste relevant sections from the manuscript (include quotes in quotation marks "like this" to indicate direct quotes from your manuscript), or elaborate on this item by providing additional information not in the ms, or briefly explain why the item is not applicable/relevant for your study

"No financial or material support of any kind was received for the work described in this article."

X27) Conflicts of Interest (not a CONSORT item)

X27-i) State the relation of the study team towards the system being evaluated

In addition to the usual declaration of interests (financial or otherwise), also state the relation of the study team towards the system being evaluated, i.e., state if the authors/evaluators are distinct from or identical with the developers/sponsors of the intervention.

	1	2	3	4	5	
subitem not at all important	○	○	○	○	◉	essential

ล้างสิ่งที่เลือก

Does your paper address subitem X27-i?

Copy and paste relevant sections from the manuscript (include quotes in quotation marks "like this" to indicate direct quotes from your manuscript), or elaborate on this item by providing additional information not in the ms, or briefly explain why the item is not applicable/relevant for your study

"No potential conflict of interest was reported by the author(s)."

About the CONSORT EHEALTH checklist

As a result of using this checklist, did you make changes in your manuscript? *

- ○ yes, major changes
- ◉ yes, minor changes
- ○ no

What were the most important changes you made as a result of using this checklist?

The most important changes highlighted by this checklist relate to the Methods section. Although all relevant procedures were carried out as part of the study, we were not explicitly documented in the original manuscript. The checklist helped identify these missing details to ensure we are properly reported.

How much time did you spend on going through the checklist INCLUDING making changes in your manuscript *

5 days for edited item-by-item

As a result of using this checklist, do you think your manuscript has improved? *

- (selected) yes
- no
- อื่นๆ:

Would you like to become involved in the CONSORT EHEALTH group?

This would involve for example becoming involved in participating in a workshop and writing an "Explanation and Elaboration" document

- (selected) yes
- no
- อื่นๆ:

ล้างสิ่งที่เลือก

Any other comments or questions on CONSORT EHEALTH

No comment, we can follow the checklist to revise the manuscript.

STOP - Save this form as PDF before you click submit

To generate a record that you filled in this form, we recommend to generate a PDF of this page (on a Mac, simply select "print" and then select "print as PDF") before you submit it.

When you submit your (revised) paper to JMIR, please upload the PDF as supplementary file.

Don't worry if some text in the textboxes is cut off, as we still have the complete information in our database. Thank you!

Final step: Click submit !

Click submit so we have your answers in our database!

ส่ง ล้างแบบฟอร์ม

[illegible]

[illegible]

[illegible] รายงาน

Google ฟอร์ม
